# A cre-inducible *DUX4* transgenic mouse model for investigating facioscapulohumeral muscular dystrophy

**DOI:** 10.1371/journal.pone.0192657

**Published:** 2018-02-07

**Authors:** Takako Jones, Peter L. Jones

**Affiliations:** 1 Department of Pharmacology, Center for Molecular Medicine, University of Nevada, Reno School of Medicine, Reno, Nevada, United States of America; 2 Department of Cell and Developmental Biology, University of Massachusetts Medical School, Worcester, Massachusetts, United States of America; University of Minnesota Medical Center, UNITED STATES

## Abstract

The Double homeobox 4 (*DUX4*) gene is an important regulator of early human development and its aberrant expression is causal for facioscapulohumeral muscular dystrophy (FSHD). The DUX4-full length (*DUX4-fl*) mRNA splice isoform encodes a transcriptional activator; however, DUX4 and its unique DNA binding preferences are specific to old-world primates. Regardless, the somatic cytotoxicity caused by DUX4 expression is conserved when expressed in cells and animals ranging from fly to mouse. Thus, viable animal models based on DUX4-fl expression have been difficult to generate due in large part to overt developmental toxicity of low DUX4-fl expression from leaky transgenes. We have overcome this obstacle and here we report the generation and initial characterization of a line of conditional floxed DUX4-fl transgenic mice, *FLExDUX4*, that is viable and fertile. In the absence of cre, these mice express a very low level of *DUX4-fl* mRNA from the transgene, resulting in mild phenotypes. However, when crossed with appropriate cre-driver lines of mice, the double transgenic offspring readily express DUX4-fl mRNA, protein, and target genes with the spatiotemporal pattern of nuclear cre expression dictated by the chosen system. When cre is expressed from the ACTA1 skeletal muscle-specific promoter, the double transgenic animals exhibit a developmental myopathy. When crossed with tamoxifen-inducible cre lines, DUX4-mediated pathology can be induced in adult animals. Thus, the appearance and progression of pathology can be controlled to provide readily screenable phenotypes useful for assessing therapeutic approaches targeting DUX4-fl mRNA and protein. Overall, the *FLExDUX4* line of mice is quite versatile and will allow new investigations into mechanisms of DUX4-mediated pathophysiology as well as much-needed pre-clinical testing of DUX4-targeted FSHD interventions *in vivo*.

## Introduction

Facioscapulohumeral muscular dystrophy (FSHD) is caused by epigenetic dysregulation of the 4q35 D4Z4 macrosatellite repeat, which leads to aberrant myogenic expression of the *Double homeobox 4* (*DUX4*) gene. *DUX4* encodes a paired homeobox domain transcriptional activator and regulator of cleavage-stage genes and therefore is not typically expressed in healthy somatic cells [1–10]. In FSHD, however, *DUX4* misexpression in differentiated skeletal muscle ultimately initiates numerous potentially detrimental events including the induction of apoptosis [11–14], and activation of the inflammatory immune response [15, 16].

While several *DUX4* alternative mRNA isoforms are generated by alternate 5’splice site usage in the first exon, only the *DUX4-full length* (*DUX4-fl*) mRNA is pathogenic [4, 5, 17]. *DUX4-fl* expression is highly regulated and apparently restricted to testis and cleavage-stage embryos where the DUX4-mediated transcriptional program is key for human zygotic genome activation, supporting a normal function for DUX4-FL in germ cells and during early human development [1, 3, 5, 18, 19]. However, individuals meeting the genetic, epigenetic, and clinical criteria for FSHD express stable DUX4-fl mRNA and DUX4-FL target genes in their skeletal muscles, initiating a detrimental cascade of events which culminate in muscle pathology and disease [4–6, 9, 10, 20, 21]. Somatic expression of *DUX4-fl* mRNA *per se* is not necessarily pathogenic since rare expression can be detected in some cultures of healthy myogenic cells and muscle biopsies, albeit at levels significantly lower than those found in similar tissues from FSHD-affected subjects, suggesting that the levels and/or timing of somatic DUX4 expression dictate disease [6–8]. Thus, aberrantly increased *DUX4* expression is the primary mediator of FSHD pathophysiology, the DUX4-fl mRNA and protein are prime FSHD therapeutic targets, and animal models for FSHD should be based on expression of *DUX4*.

FSHD is an autosomal dominant, gain-of-function disease and thus should be amenable to modeling in mice by overexpression of *DUX4-fl* mRNA. However, animal models of FSHD have suffered from serious issues with respect to pathogenic expression levels, tissue distribution, and cellular toxicity [12, 22–26]. Early attempts to model the disease in mice using viral over-expression produced massive pathology and physiological effects not characteristic of FSHD [13], resulting in conclusions based on artifacts [27]. Conversely, the D4Z4-2.5 transgenic mouse model, in which a pathogenic FSHD1-sized D4Z4 repeat array consisting of 2.5 D4Z4 repeat units [28, 29], including the distal *DUX4* polyadenylation site rendering the distal repeat unit capable of producing the stable *DUX4-fl* mRNA [4], suffered from too little *DUX4* expression in muscle and failed to show an FSHD-like phenotype [23]. When levels of the FSHD modifier gene, *Smchd1*, were decreased, the levels of *DUX4-fl* were increased and the phenotypes were exacerbated, however, the model still failed to recapitulate FSHD-like muscle pathology [26]. Interestingly, a tetracycline regulated transgenic knock-in *DUX4* mouse model that suffered from severe pathology and early lethality was recently rescued by inserting an inefficient *DUX4* mRNA polyadenylation signal to decrease leaky DUX4-fl transgene expression allowing for animals to survive and develop FSHD-like phenotypes in muscles [24, 30]. Together, these cases highlight both the importance and difficulty of mimicking the pathogenic mechanisms of FSHD in mouse models [31].

In FSHD, the *DUX4* gene is typically expressed in only a small fraction (<1%) of myogenic cells, ultimately leading to debilitating muscle pathology over time [5, 6, 32]. This may account, in part, for the generally late onset of clinical symptoms in FSHD patients. In addition, DUX4-fl is expressed in sporadic bursts in differentiated FSHD muscle cells, an event that is epigenetically suppressed in healthy and asymptomatic subjects [7, 14, 33]. However, DUX4-fl expression in affected FSHD muscle, even when bursting, is still extremely rare, highly variable, and difficult to detect [5, 6, 14]. Thus, based on our current understanding of the FSHD pathogenic mechanism, adult-onset, mosaic transgene expression in skeletal muscle is necessary for modeling an FSHD-relevant DUX4 expression profile and is key to achieving FSHD-like pathophysiology. Yet nothing is known about potential developmental or extra-muscular contributions of DUX4-fl expression to FSHD. Ideally, a mouse model for investigating this disease should incorporate flexibility in dictating DUX4-fl expression, and thus allow some of these critical questions to be addressed.

Here we report the generation and initial characterization of a conditional DUX4-fl transgenic mouse model, termed *FLExDUX4*. Similar to the situation in FSHD, *DUX4-fl* is expressed at extremely low levels in adolescents and adult *FLExDUX4* mice. Mating with cre-expressing lines of mice leads to induction of DUX4-fl in the spatiotemporal manner of the cre driver, thereby providing a highly flexible model for investigating the effects of DUX4-fl expression. When crossed with a skeletal muscle-specific, tamoxifen inducible cre-expressing mouse strain, skeletal muscles of the double transgenic offspring can be induced to produce mosaic expression patterns of *DUX4-fl* expression in a fraction of skeletal myonuclei, similar to the spontaneous mosaic bursting of DUX4-fl expression found in FSHD skeletal myocytes [14], ultimately resulting in an FSHD-like myopathy. Thus, *FLExDUX4* mice are a useful model for both developmental and disease-relevant studies on DUX4-fl, and provide a suitable model for FSHD therapeutic interventions targeting DUX4-fl mRNA, protein, and certain downstream pathways.

## Materials and methods

### Ethics statement

All animal procedures were approved by the local IACUC committees at the University of Massachusetts Medical School and the University of Nevada, Reno. The work performed at the University of Nevada, Reno was approved under protocol #0701. The work performed at the University of Massachusetts Medical School was approved under protocols #A-2447-13 and #A-2535-16. Anesthesia was performed using isoflurane inhalation. Euthanasia was performed using isoflurane inhalation followed by exsanguination.

### Transgenic mouse generation and crosses

Transgenic mice were generated by genOway SA (France) using the FLEx directional switch system to bypass the embryonic lethality from leaky embryonic *DUX4* transgene expression [34, 35]. The *DUX4* genomic sequence (S1 Sequence), containing all three exons and both introns, was synthesized *in vitro* with silent mutations made into the two known 5’ splicing donor sites for the *DUX4-s* isoforms. This sequence was cloned into the proprietary genOway *Rosa26* targeting vector, and completely sequenced. Murine C57BL/6 ES cells were transfected and clones were selected and screened for homologous recombination into the *Rosa26* locus, first by PCR, then confirmed by Southern blotting. Properly targeted ES cells were injected into blastocysts and implanted to generate male chimeras. The chimeras were mated to C57BL/6 females to confirm germline transmission. Chimeras were mated with the genOway proprietary ubiquitous Flp recombinase expressing mice for in vivo removal of the Neomycin selection cassette. The F1 hemizygous *FLExDUX4* mice devoid of the Neomycin resistance gene were delivered to the Jones lab.

C57BL/6 mice and the following Cre driver lines were purchased from Jackson Labs (Bangor, Maine): ACTA1-MCM refers to B6.Cg-Tg(ACTA1-cre/Esr1*)2Kesr/J (JAX 025750), Sox2-cre refers to B6.Cg-Tg(Sox2-cre)1Amc/J (JAX 008454), ACTA1-cre refers to B6.Cg-Tg(ACTA1-cre)79Jme/J (JAX 006149), UBC-cre/ERT2 refers to B6.Cg-Tg(UBC-cre/ERT2)1Ejb/J (JAX 008085), and Pax7-cre/ERT2 refers to B6.Cg-Pax7<tm1(cre/ERT2)Gaka>/J (JAX 017763).

### Transgenic mouse genotyping

Genomic DNA was isolated from 2mm tail snips from <10-day-old mice. PCRs were performed with ~100ng genomic DNA, 1X GoTaq Buffer, 200μM dNTPs, 0.5U GoTaq DNA polymerase (Promega), 600nM primer TJ76F (5’-CAATACCTTTCTGGGAGTTCTCTGCTGC), 400nM primer TJ77R (5’- CTCGTGTAGACAGAGCCTAGACAATTTGTTG), 200nM primer TJ78R (5’- TGCAGGACAACGCCCACACACC). Reactions were incubated at 94°C for 3min, then cycled 30 times (94°C for 20sec, 62°C for 20sec, 72°C for 35sec), followed by a final 2min extension at 72°C. Reactions were separated on a 1.5% agarose gel and visualized by ethidium bromide staining. Homozygous pups produce a 409bp product, hemizygous pups produce 409bp and 175bp products, and wild type pups produce a 175bp product.

### Transgene recombination

Genomic DNA was isolated from the organic phase of TRIzol RNA tissue extractions as per manufacturer’s protocol. Two PCRs were performed on genomic DNA (50ng/reaction) using 0.1μl GoTaq (Promega) in 1X GoTaq buffer, 200μM dNTPs, 400nM primer TJ76F (5’-CAATACCTTTCTGGGAGTTCTCTGCTGC), and either 400nM reverse primer TJ77R (5’-CTCGTGTAGACAGAGCCTAGACAATTTGTTG) for detecting the non-recombined transgene (406bp product) or 400nM reverse primer 14A2-Rev (5’-AGGCTCGCAGGGCCTCGCTT) for detecting the recombined transgene (440bp product). Reactions were incubated at 94°C for 3min, then cycled 32 times (94°C for 20sec, 62°C for 20sec, 72°C for 35sec), followed by a final 2min extension at 72°C. Reactions were electrophoresed in separate wells of the same 1.5% agarose gel and visualized by ethidium bromide staining. Band intensities were quantified and the recombination rate was calculated as % recombination = TJ76F;14A2-Rev band / (TJ76F;TJ77R band + 76F;14A2-Rev band) x 100.

### Tamoxifen (TMX) administration

For intraperitoneal (IP) injections of TMX, tamoxifen free base (Sigma #T5648) was dissolved in 100% ethanol (200mg/ml) at 55°C and added to warm sterile corn oil (ThermoFisher S25271) to make a 20mg/ml stock. Stock TMX aliquots were warmed to 37°C, briefly, and diluted further with warm corn oil 10-fold just prior to use. Twelve-week-old mice were weighed and injected IP on two consecutive days with the appropriate volume (100μl for a 20g mouse) of TMX for a final concentration of 10mg/kg. Alternatively, when specifically indicated, TMX-laced chow (400mg/kg tamoxifen citrate) purchased from Envigo (TD.130860) was provided *ad libitum* for 5 days providing an average daily dose of ~40mg/kg for 20-25g body weight assuming a 3-4g daily food intake.

### DNA methylation analysis

Genomic DNA was isolated from dissected muscle samples using TRIzol (ThermoFisher), as per manufacturer’s instructions, bisulfite converted, and analyzed using the bisulfite sequencing 4qA (BSSA) protocol, as described [7, 36].

### Gene expression analysis by RT-PCR

Total RNA was extracted from dissected mouse muscles homogenized in 10 volumes TRIzol (ThermoFisher) using the TissueLyser LT (Qiagen), as per manufacturer’s instructions, followed by clean-up using the RNeasy mini kit (Qiagen) to remove all remaining genomic DNA. Qualitative expression of the 1.4kb *DUX4-fl* RT-PCR product was performed as described [4]. Control reactions skipping the first-strand synthesis step were performed to ensure purity of the RNA samples and assuring the results were not from contaminating genomic DNA. Quantitative *DUX4-fl* mRNA expression was analyzed using nested qRT-PCR, as described [37]. Expression of *DUX4* target genes was analyzed by qPCR using 5-50ng cDNA, generated with iScript Supermix (Bio-Rad) per manufacturer’s instructions. Expression of *DUX4-fl* was normalized to 18S rRNA or *RpL37* RNA as indicated, expression of target genes was normalized to *RpL37* RNA. Oligonucleotide primer sequences for *DUX4-fl*, *RpL37*, and *18S* rRNA are as previously reported [33]. Oligonucleotide primer sequences for *Trim36*, *Wfdc3*, and *Cxcr4* are as previously reported [23].

### Alcian blue staining

The E16.5 embryos were obtained from crossing male *ACTA1-cre/+* with female *FLExDUX4/+*, and the extraembryonic membrane of each embryo was collected and used for genotyping. Alcian blue staining of the fetal E16.5 cartilaginous skeleton was performed as described [38]. Briefly, embryos were dissected at stage 16.5 d.p.c. in PBS and the extraembryonic membrane was removed and used for genotyping. Embryos were fixed in Bouin’s fixative for 2h, washed in ammonium hydroxide:ethanol solution for 24h with 6 changes of solution and equilibrated in 5% acetic acid twice for 1hr each. Embryos were soaked in alcian blue stain for 2h, washed twice in 5% acetic acid for 1h each, then cleared in methanol (2X for 1h each) and then finally in BABB (benzyl alcohol:benzyl benzoate) solution. The stained embryos were imaged using the Leica MZ7.5 and Zeiss Axiocam.

### Histology

Freshly dissected muscles were kept moist, coated with O.C.T. compound, frozen in liquid nitrogen-cooled isopentane and stored at -80°C until sectioning. The 10–12μm cryosections were mounted on slides, air-dried for 30 min before staining or storage. Sections were fixed with cold acetone for 5 min for Hematoxylin and Eosin staining or picrosirius red staining.

### Picrosirius red staining

Staining was performed as described [39]. Cryosections (12μm) cut mid belly of the Tibialis anterior muscle or heart were mounted and fixed in 4% paraformaldehyde/PBS for 10 min, rinsed with dH_2_O, and then dehydrated with a series of 30 sec ethanol washes (70%, 95%, 100%) and air dried. Sections were stained for 1 hr in fresh picrosirius red solution (0.1% direct red 80, 1.3% saturated picric acid), washed twice with 0.5% acetic acid and three times with dH_2_O. Stained sections were dehydrated with a series of ethanol washes (70% for 30 sec, 95% for 30 sec, and 100% for 1 min), cleared with xylene for 5 min, and mounted with Cytoseal 60. A series of micrographs from each muscle section were captured using a 10X objective on a Leica DM2000 and reconstituted to form an entire muscle cross-section. A custom script was written in MATLAB (Mathworks) to determine the number of pixels stained red and the total number of pixels stained. Muscles from 3 mice, 5 sections per muscle, for each treatment were analyzed.

### DUX4 Immunohistochemistry

Tissue sections were fixed with 4% paraformaldehyde/PBS on ice for 20 min, permeabilized with 0.25% TritonX-100/PBS for 10 min, then incubated with blocking solution (5% normal goat serum (NGS), 2% BSA, 0.01% TritonX-100/PBS) for 30 min. Sections were incubated with DUX4 E5-5 antibody (1:200, abcam) at 4°C overnight, then subsequently incubated with Alexa 594 Goat anti-rabbit IgG (1:500, ThermoFisher) at room temperature for 40 min. Stained sections were mounted in ProLong Gold with DAPI for nuclear staining.

### Four-limb hanging test

A 6x6-inch wire mesh made of 2-mm thick stainless steel wire was hand-held ~35-cm above a layer of soft bedding material. Mice were allowed to grasp the middle of the mesh in an upright position and acclimated. The mesh was slowly rotated until inverted, the timer was started, and time until the mouse fell to the bedding was recorded. Each mouse was given three trials per session and the longest hang time was recorded.

## Results

### Generation and Characterization of the *FLExDUX4* mouse line

**Fig 1:**
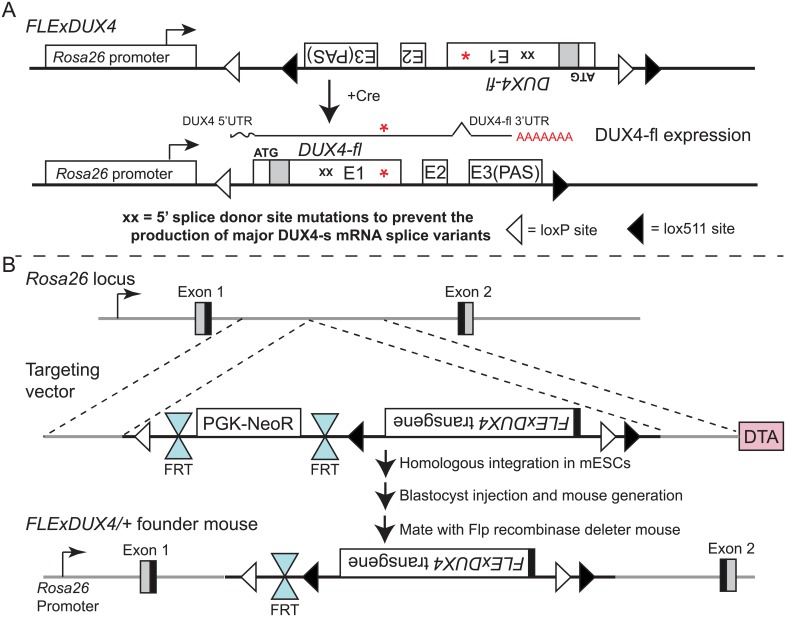
Transgene map and transgenic model generation. (A) The synthesized FLExDUX4 transgene, which maintains the 3 exon and 2 intron structure of the human *DUX4* gene, but lacks the *DUX4* promoter, was inserted in the antisense orientation to the *Rosa26* promoter. The transgene is flanked by heterologous lox sites (loxP and lox511) that unidirectionally recombine in the presence of cre recombinase to invert the FLExDUX4 transgene to the sense orientation. *DUX4-fl* mRNA is then transcribed from the *Rosa26* promoter, processed, and terminated by the *DUX4* exon3 PAS. (B) The genome targeting scheme for knocking in to the *Rosa26* locus utilized the FLEx system methodology. PGK-NeoR: *phosphoglycerate kinase I* promoter regulating the neomycin resistance gene. DTA: *Diphtheria toxin A* gene used for counter selection.

Our goal was to generate a viable and fertile transgenic mouse model containing an intact human *DUX4* transgene that, upon controlled induction, produces mosaic expression and a myopathic phenotype [4, 10, 18]. While the DUX family of transcription factors is functionally conserved in mammals, the intact human *DUX4* gene is specific to old-world primates and not found in mice [2, 3, 19, 40]. Therefore, investigating the potential pathogenic effects of DUX4-fl expression in mice requires the human *DUX4* gene to be incorporated into the mouse genome. To accomplish this, we synthesized a *DUX4* transgene that retained the intact human *DUX4-fl* gene structure, including the 5’untranslated region (UTR), all three exons and both introns, the endogenous PAS, and the distal auxiliary elements that enhance *DUX4* mRNA cleavage and polyadenylation events (Fig 1A, S1 Sequence) [1, 5, 17, 41, 42]. Importantly, many of these elements are prime targets for sequence-based therapies [43, 44]. We incorporated four silent mutations in the 5’ splice acceptor sites for the two most prominent *DUX4* alternative mRNA isoforms found in myogenic cells. These transcripts encode the non-pathogenic DUX4-short (DUX4-S) isoforms, which have a dominant negative effect on DUX4-FL; thus, it was important to prevent their expression from our *DUX4* transgene [5]. We also chose not to add any epitope tag to the *DUX4-fl* mRNA sequence that might interfere with splicing, downstream functions of the protein, or therapeutic targeting. Following transfection of our *DUX4* expression construct into murine C2C12 cells, we confirmed its functionality by RT-PCR, and the lack of detectable expression of *DUX4-s* mRNA (S1 Fig). Finally, because DUX4-FL is highly cytotoxic during vertebrate development, resulting in past failures or extreme phenotypes of transgenic mice [12, 22–24], the transgene was engineered using the FLEx directional switch system (Fig 1B) to bypass the embryonic lethality from leaky embryonic *DUX4* transgene expression [34, 35]. The FLExDUX4 transgene targeted the *Rosa26* locus such that it would recombine in the antisense orientation. The transgene was flanked by heterologous loxP sites that recombine unidirectionally upon exposure to Cre recombinase, resulting in *DUX4-fl* mRNA expression under transcriptional control of the *Rosa26* promoter. This design resulted in the successful generation of the hemizygous *FLExDUX4* line of mice in a C57BL/6 background, formally referred to as B6(Cg)-*Gt(ROSA)26Sor*^*tm1*.1(DUX4^*^)/Plj^/J, which we have made available to the FSHD research community prior to this initial publication (Jackson Laboratories catalog #028710). The available hemizygous line has been crossed with the background mouse strain (C57BL/6) at least 5 times. DNA methylation analysis of the transgene shows no changes in the pattern of transgene hypomethylation across all five generations (S2 Fig).

**Fig 2:**
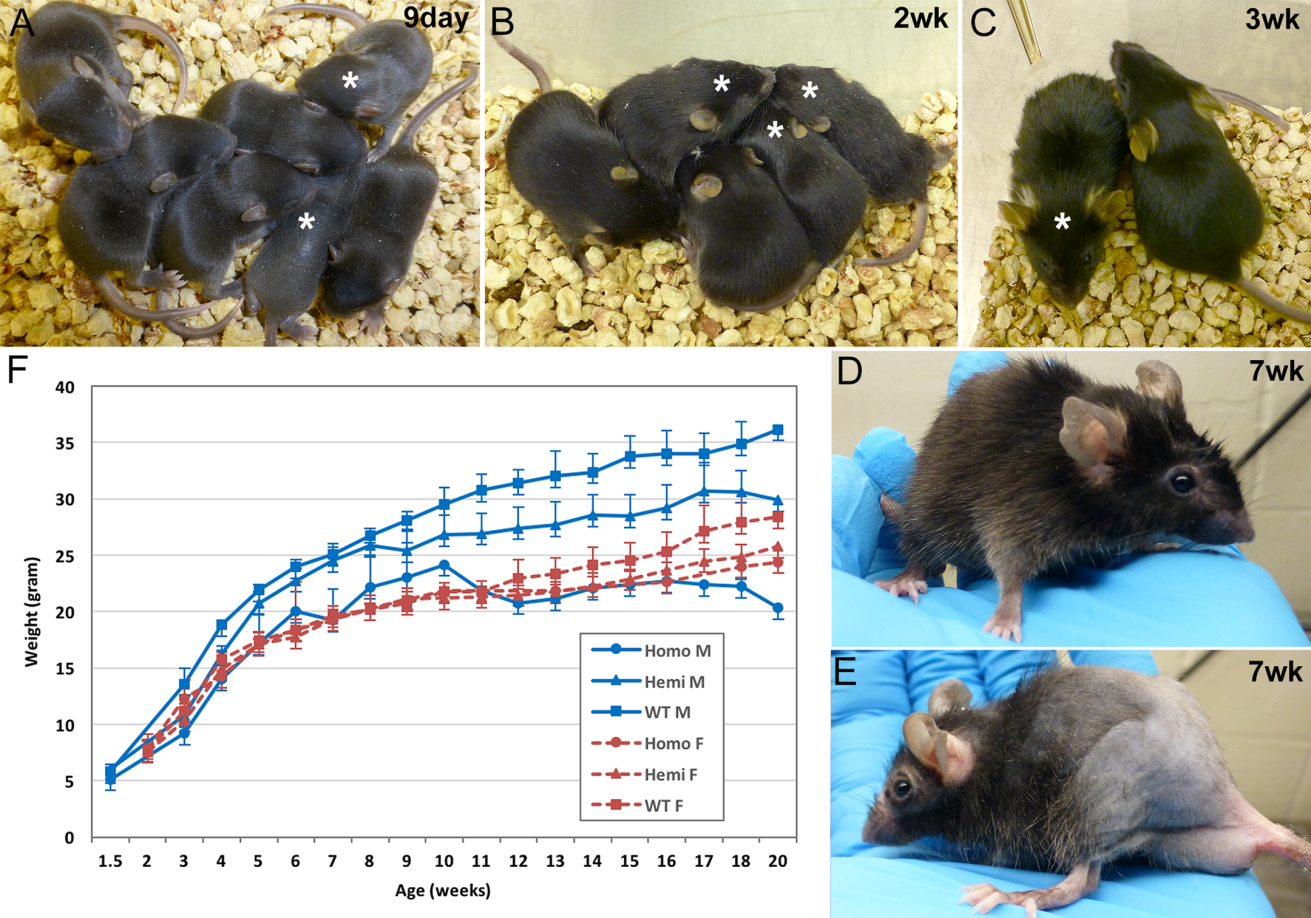
The *FLExDUX4* lines of mice. (A-D) Hemizygous (*)*FLExDUX4/+* mice are healthy, active, and fertile with a very mild alopecia that is readily distinguishable from wildtype littermates starting at two weeks of age. (E) Homozygous *FLExDUX4/FLExDUX4* mice are also healthy, active, and fertile but show more prominent alopecia than hemizygous mice. F) These transgenic mice are initially similar in size and weight as their control non-transgenic siblings; however, the hemizygous and homozygous are smaller as adults. The graph shows average weights for n = 5+ animals for each genotype measured each week for 20 weeks.

Initial observations of the *FLExDUX4/+* hemizygous mice indicated that they are viable, fertile, live a normal life span (~2yrs), and appear generally healthy with the exception of a very mild alopecia (Fig 2A–2D) and slightly reduced size (Fig 2F). Hemizygous males were similar in weight to non-transgenic C57BL/6 littermate controls through the first 8 weeks after birth, at which point the *FLExDUX4/+* males displayed slower growth, resulting in mice ~14% (~5g) smaller by 20 weeks of age. Female hemizygous mice were comparable in size to controls through 12 weeks before their weight gain slowed, resulting in ~11% (~3g) smaller adult females. Surprisingly, despite their more normal size and weight, alopecia becomes more severe in adult female hemizygous mice than in their male counterparts (S3 Fig).

*FLExDUX4/FLExDUX4* homozygous mice were similarly viable and fertile, but showed a much more severe alopecia compared with hemizygous and non-transgenic littermates, that periodically appears between 3–8 weeks of age and then remains in older animals (Fig 2E and S4 Fig). Most *FLExDUX4* homozygous mice lived a normal life span, although some developed rectal prolapse after 6 months of age and had to be euthanized. In addition, the homozygous *FLExDUX4* mice had generally soft stools, and males developed an inflamed preputial gland (S4 Fig), suggesting potential inflammation in the gastrointestinal tract. Homozygous *FLExDUX4* male mice were smaller from birth compared with hemizygous and control mice, with weight gain peaking at 9 weeks of age (~24g vs ~26g *FLExDUX4/+* and ~30g WT) and plateauing between 21-23g for the next 8 weeks, resulting in adults ~43% smaller than WT and 33% smaller than hemizygous littermates (Fig 2F). Interestingly, unlike the males, female homozygous *FLExDUX4* mice were similar in size to their hemizygous littermates through the first 20 weeks. All hemi- and homozygous mice appeared active and healthy, eating and drinking normally, similar to their control non-transgenic littermates, indicating that these changes are not due to reduced caloric intake. The reason for the gender-specific effect on size is not known. Together, these transgene-linked phenotypes with apparent transgene dosage effects led us to suspect that the *DUX4* transgene was being expressed at very low levels in the absence of cre-mediated recombination, despite the gene being in the antisense orientation to the *Rosa26* promoter.

**Fig 3:**
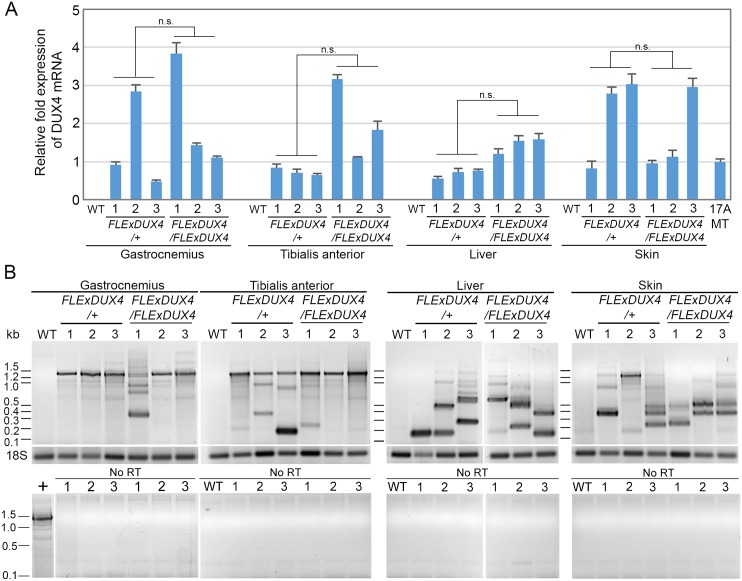
Analysis of *DUX4-fl* mRNA expression in *FLExDUX4* hemizygous and homozygous mice reveals tissue-specific transgene splicing. (A) qRT-PCR analysis of polyadenylated *DUX4* mRNA from gastrocnemius, tibialis anterior, liver, and skin isolated from hemizygous and homozygous single transgenic FLExDUX4 mice. Results of three individual mice for each genotype are reported, normalized to 18S rRNA. Significance was calculated using Welch’s t-test and the Mann-Whitney unpaired t test; n.s. = not significant (p>0.05) using either test. (B) Qualitative RT-PCR spanning exons 1–3 for *DUX4-fl* shows that skeletal muscles predominantly express the pathogenic 1.4kb and 1.2 kb *DUX4-fl* mRNAs; liver and skin predominantly express aberrant short RNAs produced from the *DUX4* transgene. Upper panels, RT-PCR utilizing oligo-dT primed first strand synthesis. Lower panels, PCR without first-strand synthesis shows no genomic DNA contamination.

To assess potential leaky expression of the *DUX4* transgene, we performed sense strand-specific quantitative reverse transcriptase PCR (qRT-PCR) for polyadenylated *DUX4-fl* mRNA using RNA extracted from several tissues of *FLExDUX4* hemi- and homozygous mice. Sense *DUX4-fl* mRNA was detected in skeletal muscle, liver, and skin of all mice (Fig 3A). Homozygous mice, on average, showed higher *DUX4-fl* expression as a group in all tissues except skin (Fig 3A), but there was mouse-to-mouse and tissue variation, and the dosage effect was not absolute or significant between groups for any tissue, despite the more severe phenotypes (S4 Fig). Regardless, the overall *DUX4-fl* mRNA levels detected were still very low, comparable to those found in differentiated human FSHD myocytes (17Abic MT [6]) (Fig 3A). To assess the scope of RNA transcript isoforms generated from the *DUX4* transgene, qualitative RT-PCR was performed using PCR primers spanning *DUX4* exons 1–3 (Fig 3B), which amplify all known *DUX4* mRNA isoforms [5]. The predominant *DUX4* transcript found in gastrocnemius and tibialis anterior muscles was the predicted 1.4 kb product for *DUX4-fl* that retains intron 1, with a minor 1.2 kb product in which intron 1 was spliced out, as is the case in FSHD muscle cells [5, 6, 45]. DNA sequencing confirmed that the 1.4 kb product is derived from the transgene, contains the conserved splicing site changes, and is expected to encode the full open reading frame for the pathogenic *DUX4-fl* mRNA (S2 Sequence). Thus, skeletal muscle preferentially generates the pathogenic *DUX4-fl* transcript, however, strikingly, both liver and skin predominantly expressed many shorter RNAs distinct from the previously described *DUX4-s* 0.35 kb and 0.45 kb isoforms [5, 6], and little to none of the 1.4 kb *DUX4-fl* transcript (Fig 3B). The nature of these aberrant transcripts is not clear, however, since the first strand cDNA synthesis was performed with an oligo-dT primer, the negative controls resulted in no amplified products (Fig 3B), and similarly the non-transgenic controls showed no products of any size, all of these isoforms most likely represent polyadenylated isoforms or truncations of *DUX4* transgene transcripts. DNA sequence analysis of these short products confirmed they are in fact derived from the transgene suggesting that *FLExDUX4* mice express novel short *DUX4* transgene transcripts in non-muscle tissues (Fig 3B). Although our approach for the efficient expression of the DUX4-fl isoform was successful in skeletal muscle, there may be tissue-specific RNA processing of the *DUX4* transgene in non-muscle tissues, which utilize variable 5’splicing donor sites and likely affects the specificity of our *DUX4-fl* qRT-PCR assay. It should be noted that the *DUX4* qRT-PCR assay was designed and validated as specific for the *DUX4-fl* mRNA in human muscle tissue and myogenic cells, not in skin or liver, since *DUX4-fl* is not known to be expressed in these tissues [7, 46]. Thus, one should interpret results with caution when using this assay alone as a measure for *DUX4-fl* mRNA levels in non-myogenic tissues of this mouse model.

To determine if this unexpected transgene expression was due to aberrant recombination, genomic PCRs for the sense and antisense transgene were performed and no indication of any genomic recombination was found (S5 Fig), thus confirming that the mRNAs are produced from antisense transcription at the *Rosa26* locus. We conclude that a sense polyadenylated *DUX4-fl* mRNA is being produced in skeletal muscle from the inverted transgene (Fig 1A) by antisense transcription at the *Rosa26* locus. Thus, in the absence of induction, *FLExDUX4* hemi- and homozygous mice have a very low level of *DUX4-fl* mRNA in their skeletal muscles throughout their lifetime, yet exhibit no overt muscle phenotype. This low chronic *DUX4-fl* expression without any induction may represent a useful model for testing therapies targeting *DUX4-fl* mRNA.

**Fig 4:**
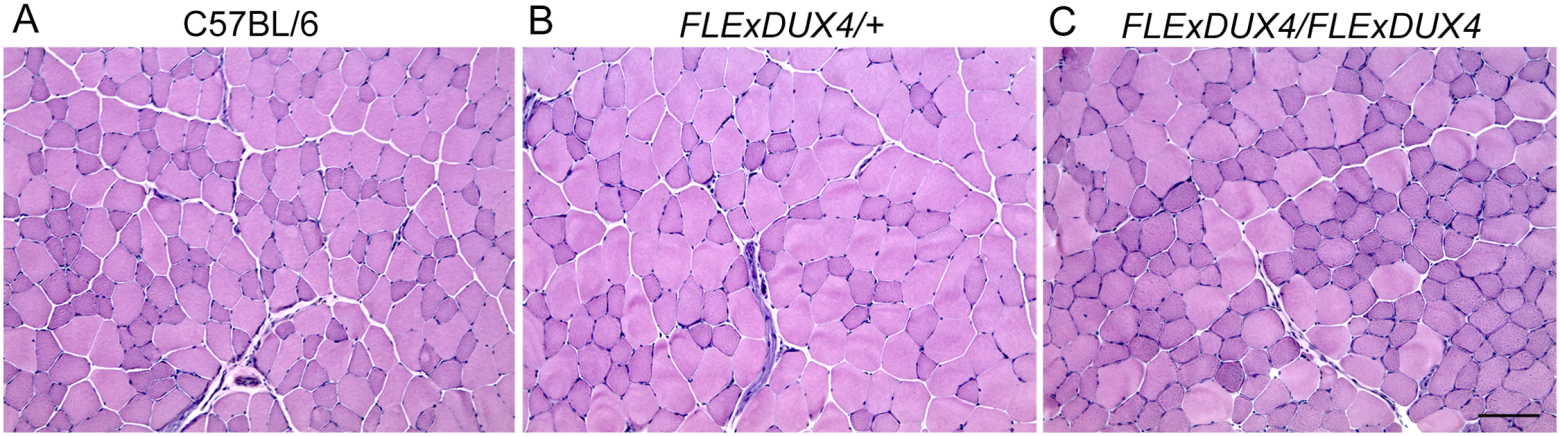
*FLExDUX4* mice display no overt myopathy despite low-level *DUX4-fl* expression. Skeletal muscles were analyzed for histopathology using hematoxylin and eosin (H&E) staining at 8, 12, 15, and 23 weeks (shown). Representative tibialis anterior sections are shown for (A) control C57BL/6, (B) *FLExDUX4/+*, and (C) *FLExDUX4/FLExDUX4* female mice. Scale bar = 100μm.

Since hemi- and homozygous *FLExDUX4* mice express low levels of *DUX4-fl* mRNA in skeletal muscles, we analyzed their muscles for DUX4-FL-mediated expression profiles and signs of histopathology. As anticipated, since there was no transgene recombination, DUX4-FL immunostaining of *FLExDUX4/+* skeletal muscles did not reveal DUX4-FL positive myonuclei (S6 Fig), indicating that the *DUX4-fl* mRNA expression seen in the muscles of these mice is likely due to very low-level expression from many or all myonuclei. DUX4-FL protein functions as a transcriptional activator in human and mice [23, 47]; to assess if functional levels of DUX4-FL protein were being made, we analyzed expression of WAP four-disulfide core domain 3 (*Wfdc3*), a well-documented direct murine target of overexpressed DUX4-FL protein [18, 23, 27], in gastrocnemius muscle (S7 Fig). Interestingly, *Wfdc3* mRNA levels were not significantly induced in either the hemi- or homozygous *FLExDUX4* muscle tissue (S7 Fig), indicating that either no protein was being made or, more likely, that protein levels in the individual myonuclei were too low to affect downstream gene transcription. Histological evaluation of skeletal muscles similarly failed to reveal any increase in centralized nuclei, immune cell infiltration, or fibrosis between hemi- and homozygous *FLExDUX4* mice and C57BL/6 controls (Fig 4 and S8 Fig). Together, this data supports a model of *FLExDUX4* mice producing very low levels of leaky *DUX4-fl* mRNA expression in all muscle cells, however, the expression levels are too little on a per cell basis to activate or at least detect induction of downstream DUX4 target genes. This is in contrast to the situation in FSHD, where a small number of myonuclei burst with high and ultimately pathogenic levels of DUX4-fl mRNA and protein expression, leading to downstream target gene activation [5, 14, 48]. This model can be readily scaled and may be useful for assessing therapeutic approaches targeting *DUX4-fl* mRNA *in vivo*.

### Developmental induction of *DUX4-fl* expression is lethal.

While *FLExDUX4* hemi- and homozygous mice express very low levels of *DUX4-fl* and display mild phenotypes in fur, digestive tract, and muscle fiber type, they show no signs of a skeletal myopathy and are generally healthy. In order to generate a model suitable for investigating DUX4-induced myopathy, it was necessary to induce DUX4-fl expression levels using cre-mediated recombination. As designed, cre expression in the tissues of double transgenic mice would lead to inversion of the transgene to the sense orientation such that *DUX4-fl* mRNA expression would be mediated by the ubiquitous and fairly strong *Rosa26* promoter [49] (Fig 1A). To assess the functionality of the floxed transgene design and ensure that cre-mediated recombination leads to increased expression of *DUX4-fl* mRNA, we initially crossed the *FLExDUX4* mice with two developmentally regulated cre expression lines of mice (Table 1). Crossing with a *Sox2-cre* mouse [50], in which cre is expressed from the *Sox2* (SRY-box containing 2) promoter in the mouse epiblast, resulted in decreased litter sizes and no double transgenic mice after multiple matings, supporting previous work that *DUX4-fl* expression during early vertebrate development is lethal [12].

**Table 1:** Results of *FLExDUX4* crosses to cre expressing lines of mice.

Cre line	Phenotype of double transgenic *FLExDUX4;cre* offspring
Sox2-cre [50]	Embryonic lethal
ACTA1-cre [51]	Stillborn E19; skeletal abnormalities
UBC-creER^T2^ [52]	Viable; may develop severe ataxia and severe neurological phenotypes requiring euthanasia after 5 weeks in absence of TMX
Pax7-creER^T2^ [53]	Viable and apparently healthy in absence of TMX
ACTA1-MerCreMer [54]	Viable, very mild muscle phenotype in absence of TMX; TMX-inducible myopathic phenotype

**Fig 5:**
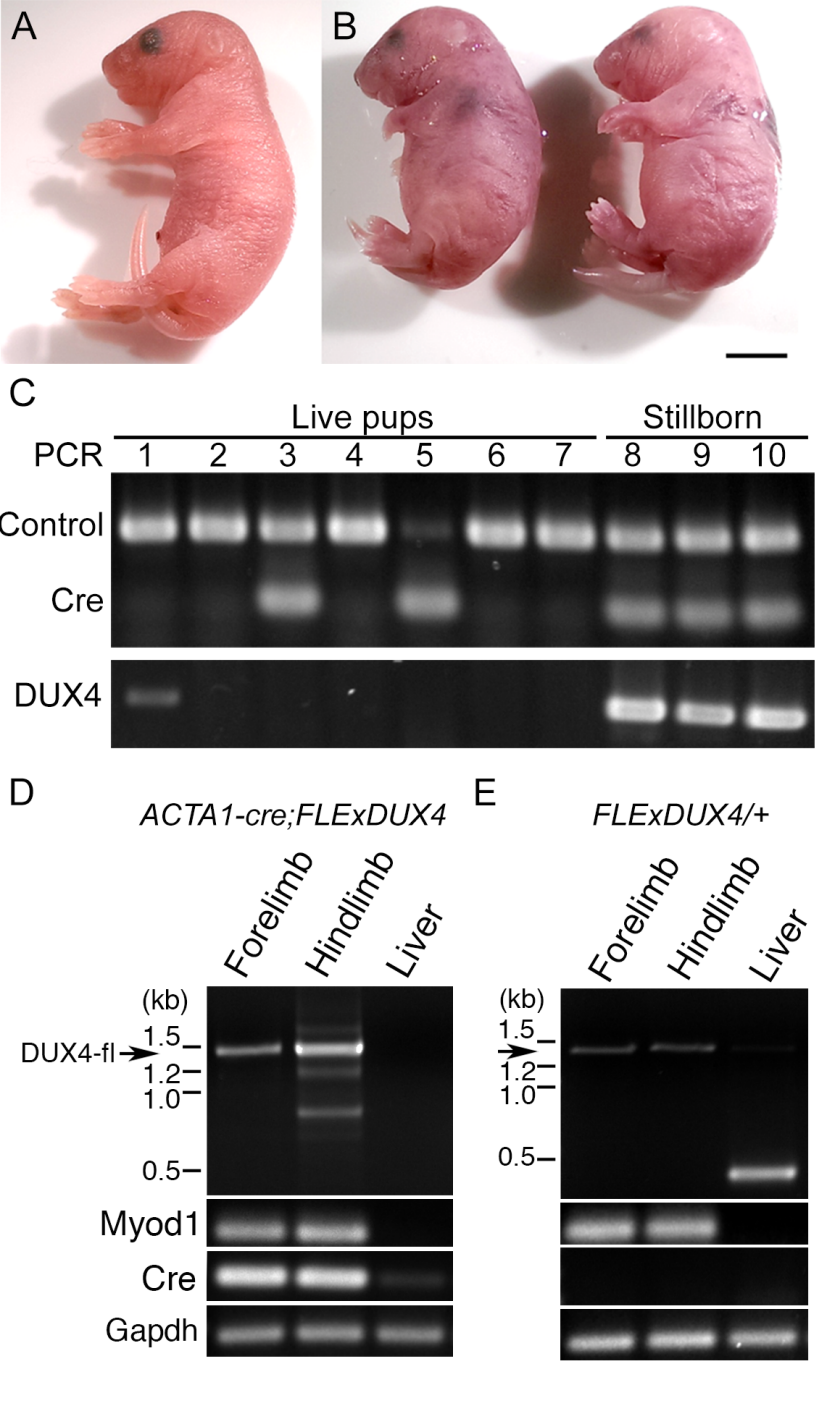
Increased expression of DUX4-fl in embryonic muscle is lethal by stage E19. In order to boost DUX4-fl expression levels in skeletal muscle, the *FLExDUX4/+* mice were crossed with *ACTA1-cre* mice. Litters were of normal size (7–10 pups) with both (A) live and (B) stillborn pups. (C) Genotyping showed live births for all three control genotypes; however, all resulting *ACTA1-cre/+; FLExDUX4/+* double transgenic animals were stillborn. D and E) RT-PCR shows that the full-length 1.4kb *DUX4-fl* mRNA transcript is expressed at increased levels in the forelimbs and hindlimbs of (D) stillborn *ACTA1-cre/+; FLExDUX4/+* animals compared with (E) live born *FLExDUX4/+* sibling pups. This increased expression correlates with expression of cre recombinase. Scale bar = 5mm.

To circumvent the developmental lethality of early ubiquitous DUX4-fl expression, *FLExDUX4* mice were crossed with *ACTA1-cre* mice [51], which express cre in striated muscles from the human *Skeletal α-actin* (*ACTA1*) promoter, recapitulating the expression profile from the endogenous murine *Acta1* promoter. Male *FLExDUX4/+* mice crossed with female *ACTA1-cre* mice and, conversely, female *FLExDUX4/+* mice crossed with male *ACTA1-cre* mice both produced litters of normal size and typical Mendelian inheritance patterns (Fig 5). However, all double transgenic pups (n = 7) were stillborn and smaller after developing nearly to full term (E19.5) compared with single transgenic or WT pups (n = 9) that were uniformly viable and healthy. RT-PCR analysis of mRNA expression confirmed that pathogenic *DUX4-fl* mRNA was expressed in all pups containing the *FLExDUX4* transgene, and at higher levels in the limbs of *FLExDUX4;ACTA1-cre* double transgenic stillborn animals compared with their *FLExDUX4/+* sibling controls (Fig 5D). Thus, while mouse development can withstand the very low level of leaky *DUX4-fl* expression found in the *FLExDUX4* hemi- and homozygous mice, increased expression levels in striated muscle during development are lethal.

**Fig 6:**
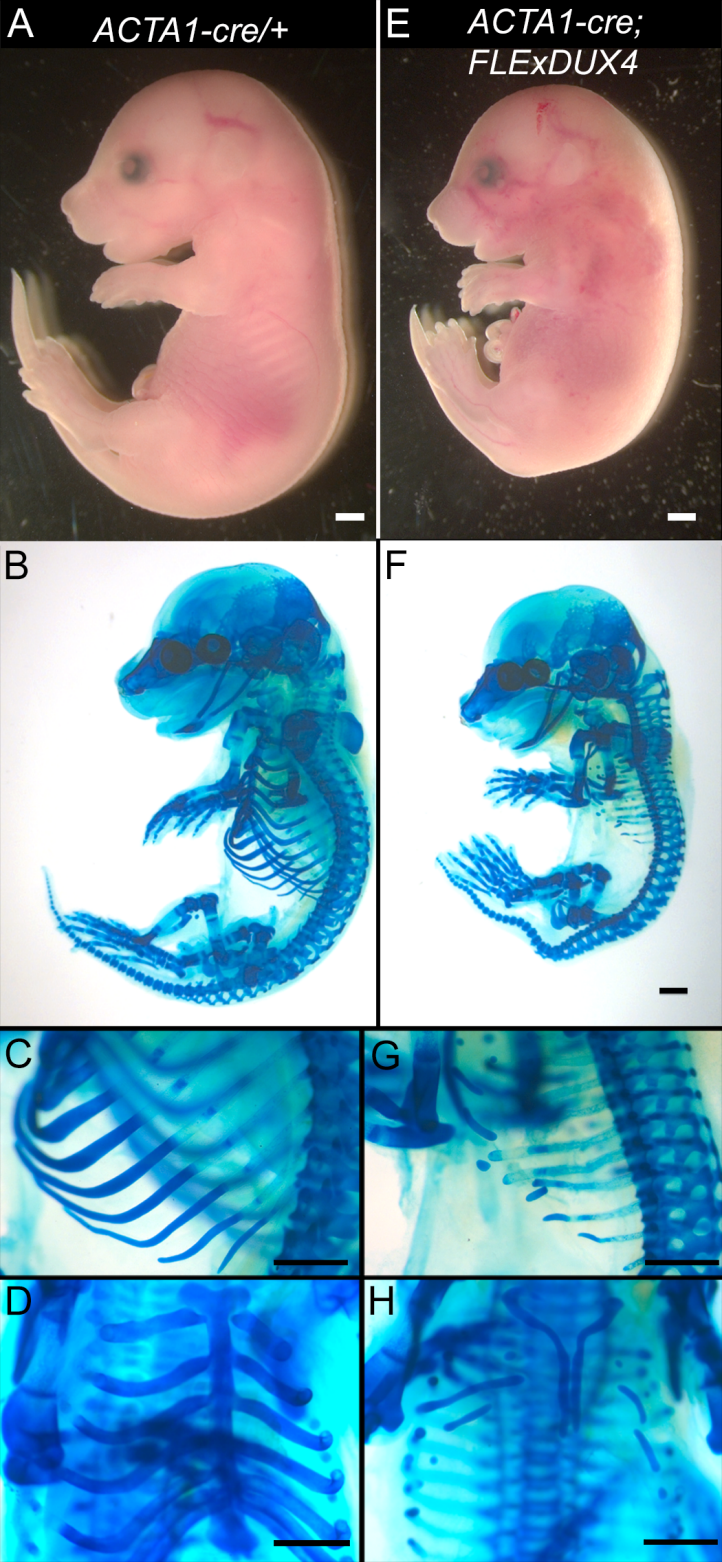
Embryonic expression of DUX4-fl results in skeletal developmental abnormalities. Stage E16.5 embryos were isolated from crosses between *ACTA1-cre/+* male and *FLExDUX4/+* female mice and stained with alcian blue to visualize the mouse fetal cartilaginous skeleton. The *ACTA1-cre/+; FLExDUX4/+* double transgenic embryos (E-H) were smaller than controls (A-D), and were the only genotype (n = 12) to show skeletal deformations in the spine, limbs, and rib cage compared with control (n = 36) littermates (*FLExDUX4/+*, *ACTA1-cre/+*, and WT). Bars = 1mm.

Although FSHD is typically adult-onset, there is an early-onset, very severe infantile form of the disease (IFSHD) that is poorly understood [55, 56], and early, potentially developmental events preceding clinical presentation of FSHD are not known. One hypothesis is that bursts of DUX4-fl expression occur earlier in IFSHD and thus start the detrimental cascade of pathophysiology sooner than in the classical adult FSHD1. An alternative and not mutually exclusive hypothesis is that all forms of FSHD have a developmental origin, establishing a course for FSHD pathology well before clinical symptoms appear. *DUX4-fl* mRNA has been detected in genetically FSHD1 human embryonic tissues [57], supporting both hypotheses and suggesting that its developmental increase in expression may be detrimental and a contributing factor to increased IFSHD severity. Since the *Acta1/ACTA1* promoter is active in the myotomal region of somites as early as 9.5 days post-coitum (d.p.c) [51], we took advantage of this developmental DUX4-fl expression model, and analyzed embryos for DUX4-fl dependent musculoskeletal defects (Fig 6). The 16.5 d.p.c. embryos were isolated from crosses of male *ACTA1-cre/+* mice with female *FLExDUX4/+* mice and analyzed with alcian blue staining to reveal the embryonic cartilaginous skeleton [38, 58]. Interestingly, all *ACTA1-cre*;*FLExDUX4* embryos (n = 12) had overall smaller bodies compared with single transgenic siblings (Fig 6A compared with Fig 6E), aberrantly developed spinal columns (Fig 6B compared with Fig 6F), and underdeveloped rib cages (Fig 6C and 6D compared with Fig 6G and 6H). None of these phenotypes were found in any sibling controls (n = 36). Thus, although these specific developmental phenotypes do not directly translate to known FSHD or IFSHD clinical phenotypes, they clearly demonstrate that small increases in DUX4-fl expression levels in striated muscle during development can have dramatic and deleterious effects.

### Inducible crosses generate phenotypic *DUX4-fl*-expressing mice

Although the *FLExDUX4* mice are viable and relatively healthy despite the presence of low levels of DUX4-fl during development, the fully penetrant stillborn phenotype of the *ACTA1-cre;FLExDUX4* double transgenic animals indicated that an adult mouse model would require postnatal induction of *DUX4-fl* expression. We therefore crossed *FLExDUX4* mice with inducible cre expressing mouse lines (Table 1). We chose three mouse lines for the crosses, each serving different purposes: induced ubiquitous expression (*UBC-cre/ERT2* line [52]), induced limb and craniofacial skeletal muscle expression (*ACTA1-MerCreMer* line [54]), and induced muscle satellite cell expression (*Pax7-cre/ERT2* line [53]). Each line expresses a cre protein fused to estrogen receptors for strong cytoplasmic retention, and mutated such that it translocates to the nucleus only in the presence of tamoxifen (TMX), and not estrogen [59]. All three crosses with *FLExDUX4* mice successfully produced litters of normal size with Mendelian inheritance, and numerous viable double transgenic offspring that survived into adulthood.

**Fig 7:**
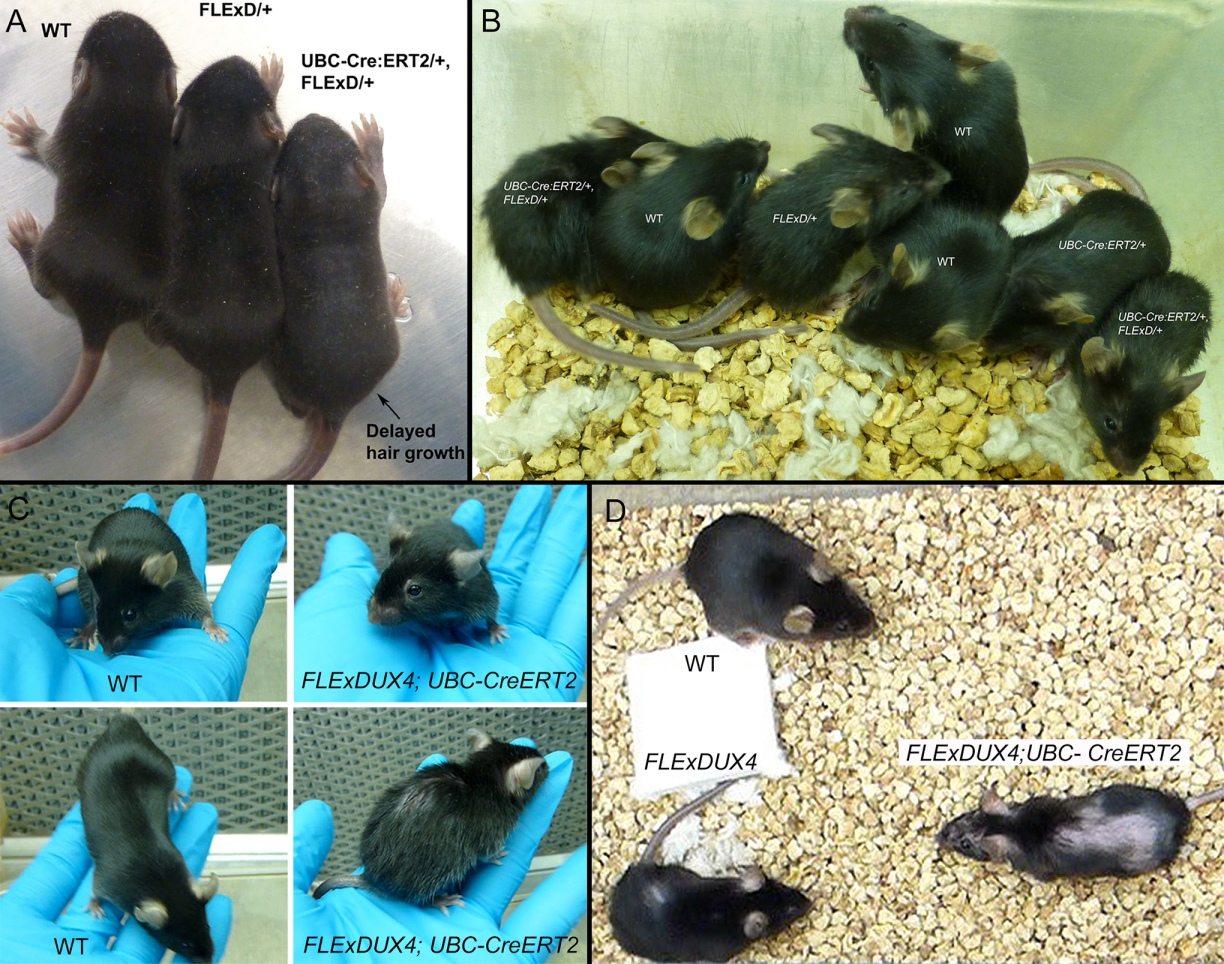
Leaky, ubiquitous *DUX4-fl* expression causes a severe adult phenotype. *FLExDUX4/+* mice were crossed with the *UBC-creERT2* line, which expresses a tamoxifen-inducible cre recombinase from the ubiquitous UBC promoter. (A) Double transgenic pups (age P10) were normal-sized, but exhibited delayed hair growth. At (B) 3 weeks of age, and (C) 6 weeks of age, double transgenic pups were still normal-sized, but had noticeably more alopecia than *FLExDUX4/+* littermates. (D) By 12 weeks, most double transgenic mice had developed severe alopecia, kyphosis, ataxia, and neurological abnormalities (S1–S3 Movies) and required euthanasia (n = 5/6; 3 male and 2 female). One male remained relatively unaffected for 16 months.

The *UBC-cre/ERT2;FLExDUX4* mice ubiquitously express a TMX-inducible cre from the human ubiquitin C promoter [52], and, in the absence of TMX administration, should not express levels of *DUX4-fl* mRNA above those caused by antisense transcription of the inverted FLExDUX4 transgene. Therefore, double transgenic animals were not anticipated to have any adverse phenotypes. However, despite apparently normal embryonic development and birthrates, the *UBC-cre/ERT2;FLExDUX4* pups (stage P10) showed delayed fur growth compared with *FLExDUX4/+* siblings (Fig 7A), which further manifested as a mild alopecia by 3 weeks of age (Fig 7B). Unlike the *FLExDUX4* hemi- and homozygous mice that remained healthy, after 5 weeks of apparently normal behavior (Fig 7C), the health of *UBC-cre/ERT2;FLExDUX4* mice began to rapidly deteriorate and they developed additional phenotypes that became much more severe (Fig 7D). Animals developed severe kyphosis and tremors, becoming unsteady and eventually ataxic (S1 Movie). Between 5–12 weeks of age, the mice (n = 5/6) had lost their righting reflex and began having spontaneous seizures characterized by periods (> 30sec) of wild and uncontrolled running (S2 Movie), and required euthanasia.

These severe phenotypes beyond those seen in the *FLExDUX4* hemi- and homozygous mice suggested increased levels of DUX4-fl expression in the double transgenic animals. Analysis of genomic DNA isolated from tissues of *UBC-cre/ERT2;FLExDUX4* mice confirmed low levels of cre-dependent transgene recombination specifically in the brain, but not in liver or muscle, and were absent in the *FLExDUX4/+* lines (S9 Fig), indicating a low level of functional nuclear cre in the brains of double transgenic mice in the absence of TMX. This leaky transgene recombination in the brain, but not the muscle, likely explains the severe neurological phenotype without an obvious muscle phenotype.

**Fig 8:**
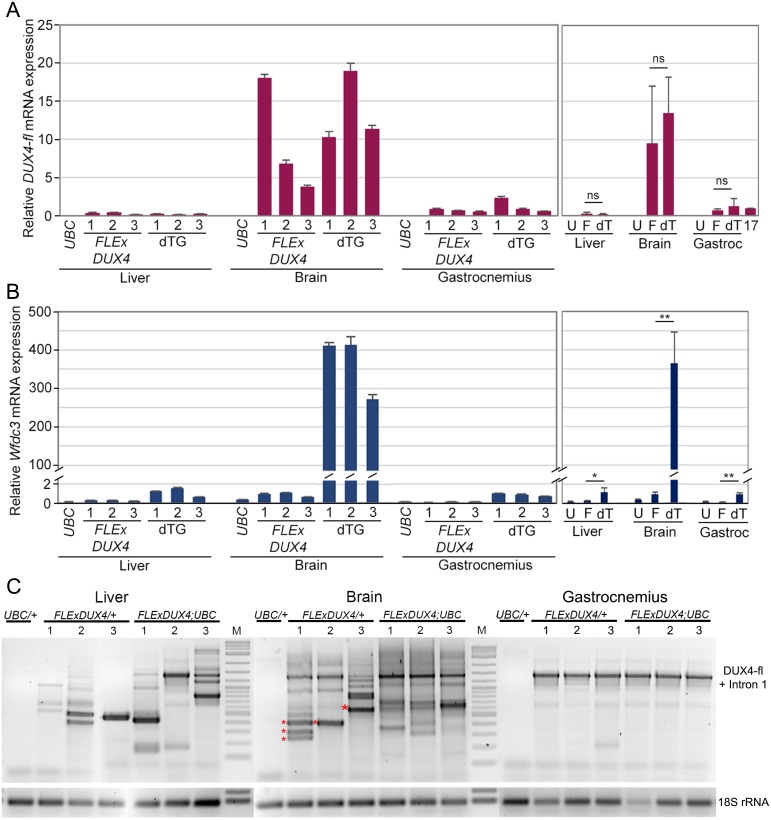
Tissue-specific *DUX4-fl* alternative mRNA isoforms are generated from the FLExDUX4 transgene. Three severely phenotypic 12-week-old *UBC-creERT2;FLExDUX4* (dTG) mice and three sibling *FLExDUX4/+* mice were analyzed by qRT-PCR for expression of (A) *DUX4-fl* and (B) *Wfdc3*, a direct DUX4-FL target gene, in liver, brain, and gastrocnemius muscle. The results of the three individual mice for each condition are summarized in the graphs on the right (U = *UBC-creERT2/+*; F = *FLExDUX4/+*; dT = dTG). (C) RT-PCR analysis of *FLExDUX4/+* and *FLExDUX4;UBC-creERT2* mice for *DUX4* mRNA isoforms shows tissue-specific splicing and variation in expression levels. Multiple novel short *DUX4* mRNAs (*confirmed by sequencing) are detected in the brain and liver, but not gastrocnemius. Significance was calculated using Welch’s t-test; n.s. = not significant (p>0.05), * = p<0.05, ** = p<0.005.

To assess the phenotypic *DUX4-fl* expression levels, we performed qRT-PCR analysis. However, the *DUX4-fl* mRNA levels detected in brain tissue from *UBC-cre/ERT2;FLExDUX4* double transgenic animals were surprisingly similar to the *DUX4-fl* expression found in the brains of non-recombined *FLExDUX4/+* mice (Fig 8A). In fact, there were no significant differences in *DUX4-fl* mRNA expression levels assayed by qRT-PCR between *FLExDUX4/+* and *UBC-cre/ERT2;FLExDUX4* mice for any of the tissues analyzed. Previous analysis of the *FLExDUX4* single transgenic mice showed that non-myogenic tissues can produce variable short *DUX4* transcripts that can affect the qRT-PCR assay, and the results may not reflect the expression level of the pathogenic DUX4-FL protein (Fig 3). To determine the levels of functional DUX4-FL protein in these tissues, expression of *Wfdc3*, a direct target of DUX4-FL protein with minimal expression in *FLExDUX4/+* mice, was analyzed and showed a markedly distinct expression pattern (Fig 8B). Despite similar *DUX4-fl* qRT-PCR profiles between mouse strains, *Wfdc3* expression was induced >250-fold in the brains of *UBC-cre/ERT2;FLExDUX4* mice compared with *FLExDUX4/+* mice, but only mildly, yet significantly, induced in liver or muscle tissues, indicating that only the double transgenic brain had high levels of functional DUX4-FL protein. To further address the discrepancy between *DUX4* mRNA expression and DUX4-FL target gene expression in the brain, we again performed a DUX4 RT-PCR to qualitatively analyze the mRNA isoforms generated from the *DUX4* transgene in these tissues from both mouse models (Fig 8C). As before (Fig 3), gastrocnemius muscle from the *FLExDUX4/+* and *UBC-cre/ERT2;FLExDUX4* mice only produced the pathogenic *DUX4-fl* mRNA isoform, containing intron 1, and did not generate any *DUX4-s* or other alternative short mRNAs from the transgene. In contrast, brains of *FLExDUX4/+* mice and, to a lesser degree, the *UBC-cre/ERT2;FLExDUX4* mice expressed a combination of *DUX4-fl* mRNA and multiple short unique transcripts that, upon DNA sequencing (Fig 8C, *), were confirmed to be novel short *DUX4* variants. Thus, alternative splicing in non-muscle tissue again contributed to a high noise for the *DUX4-fl* qRT-PCR analysis in the brain. Thus, the extreme behavioral and physical phenotypes of the *UBC-cre/ERT2;FLExDUX4* double transgenic animals can likely be explained by brain-specific leakiness of cre, leading to brain-specific transgene recombination and, ultimately increased levels of *DUX4-fl* mRNA and functional DUX4-FL protein in the brain.

Upon observation of the internal organs, the subiliac lymph nodes (central drainage) and popliteal lymph nodes (hind leg drainage) [60] were strikingly swollen in the double transgenic mice compared with C57BL/6 and *FLExDUX4* controls (S10 Fig). After weighing, both types of lymph nodes were found to be significantly enlarged in the DUX4-fl expressing *FLExDUX4/+* (4.0 mg SD = 1.641 and 1.7 mg SD = 0.427, respectively) and *UBC-creERT2;FLExDUX4* (12.5 mg SD = 8.031 and 4.4 SD = 2.935, respectively) mice compared with control mice (2.5 mg SD = 0.601 and 0.9 mg SD = 0.326, respectively) (S10 Fig). Enlarged lymph nodes can be indicative of acute inflammation, and several lines of evidence suggest inflammatory immune processes may be involved in mediating FSHD pathophysiology [9, 15, 16, 61, 62], supporting that an inflammatory immune response was stimulated by low-level DUX4-fl expression in these mice. Overall, we conclude that *UBC-cre/ERT2;FLExDUX4* animals, with or without TMX induction, express elevated levels of DUX4-FL and may be useful for evaluating certain systemic effects of DUX4-fl expression for preclinical testing of molecules targeting DUX4-fl mRNA or protein. However, due to severe neurological effects, this model did not recapitulate an FSHD-like phenotype, and the progressive severity of the phenotype with age requires utilizing young mice and administering euthanasia by 10–12 weeks of age.

### Generation of an adult FSHD-like phenotype using the *FLExDUX4* model

Our current understanding of FSHD is that the aberrantly increased DUX4-fl expression is generally restricted to skeletal muscles [33]. However, unlike other muscle diseases, in which all of the myocytes of an affected individual share the same functional deficit [63], it is estimated that <1% of FSHD myonuclei express the pathogenic *DUX4-fl* mRNA at any particular time [5, 6], and that sporadic bursts of *DUX4-fl* expression from an increased number of myonuclei may lead to pathology [14]. Affected muscle has mosaic expression, and *DUX4-fl* expressing cells are in the minority. To best recapitulate this situation in our mouse model, we crossed *FLExDUX4* mice with the *ACTA1-MerCreMer* (*ACTA1-MCM*) line [54], which expresses TMX-inducible cre in skeletal and craniofacial muscles, but not in cardiac muscle. Presumably, the resulting *ACTA1-MCM;FLExDUX4* mice would express low levels of leaky *DUX4-fl*, similar to an unaffected FSHD patient, and, upon limited TMX administration, would recombine the transgene in a fraction of myonuclei, dependent upon the amount of TMX, to produce a mosaic *DUX4-fl* expression pattern in the skeletal muscles. This would result in high levels of *DUX4-fl* expression from each recombined myonucleus, thus generating a DUX4-mediated FSHD-like myopathy.

Crossing *ACTA1-MCM* males with *FLExDUX4* females produced large litters (8–10 pups) of viable healthy double transgenic offspring at normal Mendelian ratios. Typically, researchers aim for a 100% recombination rate for the transgene and use up to 75mg/kg TMX in 5 successive intraperitoneal (IP) injections [64] to achieve this goal. However, we are interested in a much lower recombination rate to produce mosaic expression of the *DUX4* transgene and avoid lethality. To obtain our desired phenotype, we chose two methods: 1) feeding a TMX-laced chow *ad libitum* for 5 days for a predicted administration of 40mg/kg/day, and 2) direct administration of TMX using a dosage of 20mg/kg in corn oil delivered by IP injection on two successive days. All TMX-treated *ACTA1-MCM;FLExDUX4* mice and controls were monitored daily for activity and overall health. Both groups of TMX-treated *ACTA1-MCM;FLExDUX4* mice rapidly displayed phenotypes consistent with a progressive myopathy (S11 Fig, S3–S10 Movies). The mice fed TMX chow lost weight and became ataxic within 8 days of TMX exposure (S3 Movie); however, the mice did not appear to like eating the chow and food intake was lower than with untreated food. Thus, analyzing the effects of TMX induction of *DUX4-fl* was compounded by low and variable food intake and resulting weight loss, which translated to a variable and uncontrolled dosage. We conclude that TMX-laced chow is not an appropriate route of administration for this mouse model. In contrast, there was no such variable with the mice administered TMX via IP injections. In addition, concerns about the potential effects of TMX on muscle are mitigated using this IP method since the dose is low and exposure is very short due to TMX clearance [65]. By seven days post-injection, all double transgenic mice showed a noticeable decline in appearance, activity (S4–S6 Movies), and strength (S7–S10 Movies) and by nine days post-injection, they required euthanasia. By contrast, TMX-injected *FLExDUX4/+* and *ACTA1-MCM/+*, as well as non-injected *ACTA1-MCM;FLExDUX4* controls, showed no adverse effects. Qualitative movement (compare movies of control animal, S4 and S5 Movies with movie of affected animal, S6 Movie) and strength measurements by a four-limb hanging test (compare movies of control animals, S7 and S8 Movies with movies of affected animals, S9 and S10 Movies) revealed clear declines specific to the TMX-injected *ACTA1-MCM;FLExDUX4* animals (e.g. 2 min maximum assay hanging times for all controls and <2 sec for all TMX-injected *ACTA1-MCM;FLExDUX4* affected animals). Thus, TMX-injected *ACTA1-MCM;FLExDUX4* mice develop an expected FSHD-like phenotype.

**Fig 9:**
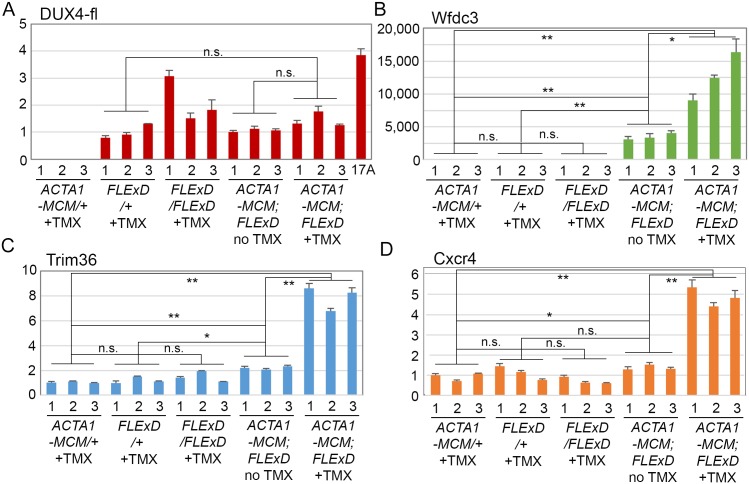
TMX induction leads to DUX4-FL target gene expression in skeletal muscle of *ACTA1-MCM;FLExDUX4* mice. Gastrocnemius muscles from 13-week-old mice were assayed 9 days post-IP injection with TMX, as indicated, and relative mRNA expression levels for *DUX4-fl* and three DUX4-FL target genes were determined. Each of the 3 individual mice per group (1, 2, 3) are shown. (A) *DUX4-fl* mRNA expression assayed by qRT-PCR does not significantly change between groups, despite increased protein expression (Fig 11). (B) *Wfdc3* mRNA expression is upregulated in both double transgenic groups compared with *FLExDUX4* alone; however, TMX treatment significantly induces *Wfdc3* expression in *ACTA1-MCM;FLExDUX4* compared with the untreated control *ACTA1-MCM;FLExDUX4* mice. In contrast, (C) *Trim36* and (D) *Cxcr4* mRNA expression are relatively constant and only upregulated in response to TMX induction in *ACTA1-MCM;FLExDUX4* mice. Significance was calculated using Welch’s t-test; n.s. = not significant (p>0.05), * = p<0.05, ** = p<0.005.

**Fig 10:**
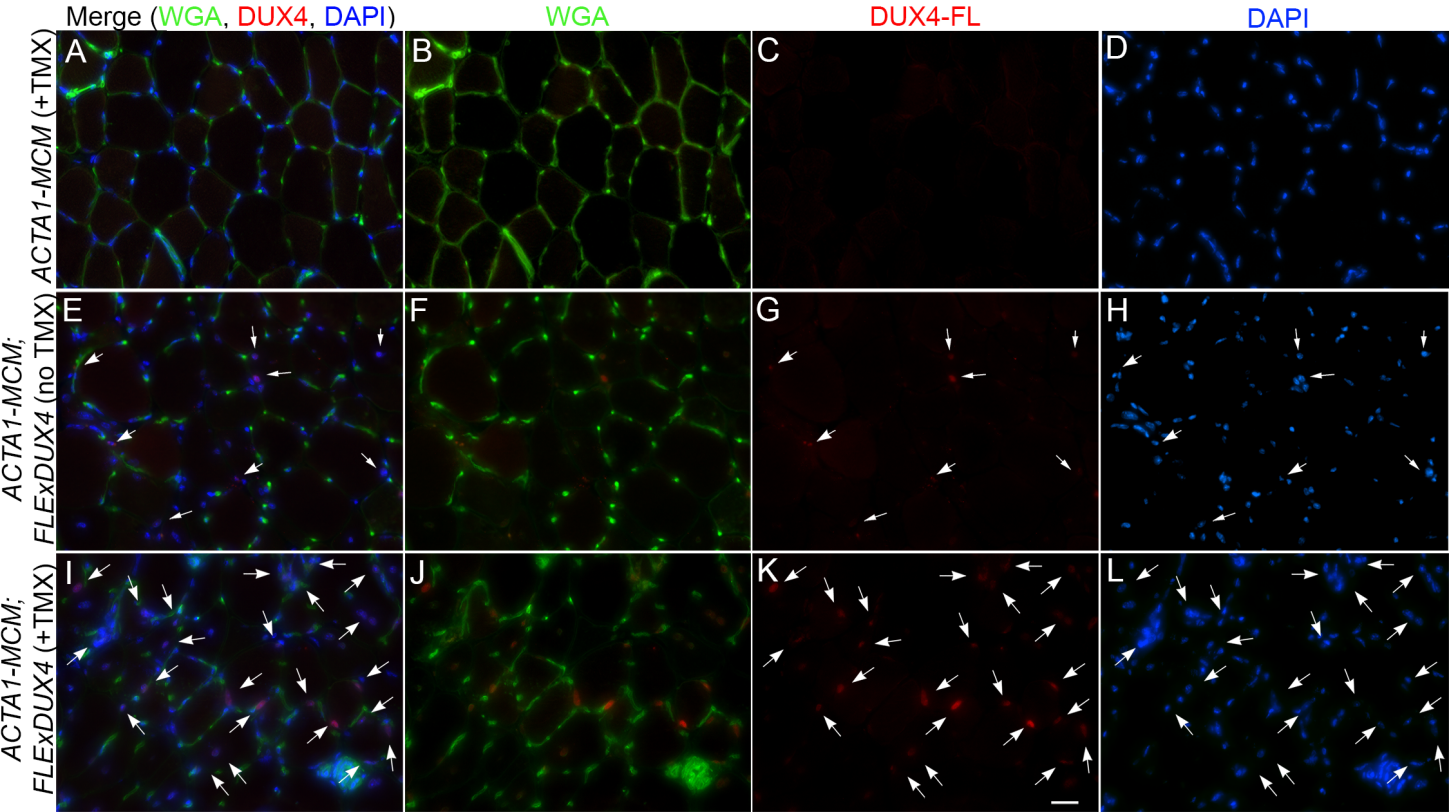
TMX induction leads to mosaic expression of DUX4-FL protein in myonuclei of *ACTA1-MCM;FLExDUX4* mice. Gastrocnemius muscles from 13-week-old mice 6 days post-IP injection with TMX, as indicated, were assayed by immunohistochemistry for DUX4-FL protein, counterstained with wheat germ agglutinin (WGA) to outline myofibers, and DAPI to identify myonuclei. (A-D) *ACTA1-MCM*, (E-H) *ACTA1-MCM;FLExDUX4* no TMX, and (I-L) *ACTA1-MCM;FLExDUX4* +TMX. Arrows identify DUX4-FL positive myonuclei. Scale bar = 25μm.

**Fig 11:**
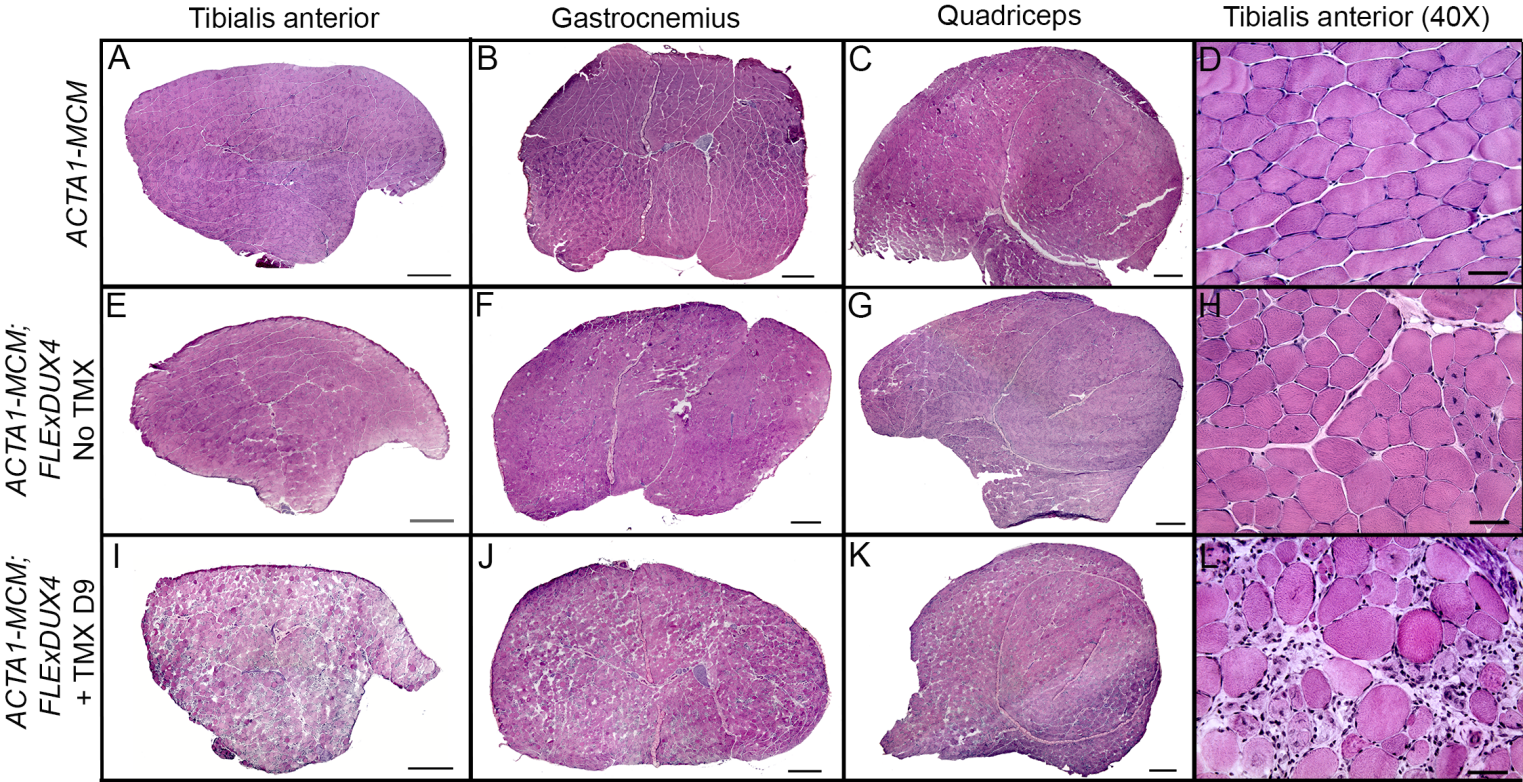
Induction of DUX4-fl induces a severe myopathy in skeletal muscles of *ACTA1-MCM;FLExDUX4* mice. H&E staining on skeletal muscle sections from 12-week-old *ACTA1-MCM/+* (A-D), *ACTA1-MCM;FLExDUX4* (E-H), and *ACTA1-MCM;FLExDUX4* +TMX mice at 9 days post-injection (I-L). The tibialis anterior (A, E, I), gastrocnemius (B, F, J), and quadriceps (C, G, K) muscles are shown at 10X (bars = 500μm), and the tibialis anterior (D, H, L) is shown at 40X (bars = 50 μm). Low level skeletal muscle transgene recombination in the absence of TMX leads to a proportion of centralized nuclei, indicative of regenerating myofibers, in *ACTA1-MCM;FLExDUX4* muscles (H). TMX induction in *ACTA1-MCM;FLExDUX4* muscles leads to increased numbers of myonuclei expressing DUX4-FL, heterogeneous fiber size distribution, and mononuclear cell infiltration (L).

**Fig 12:**
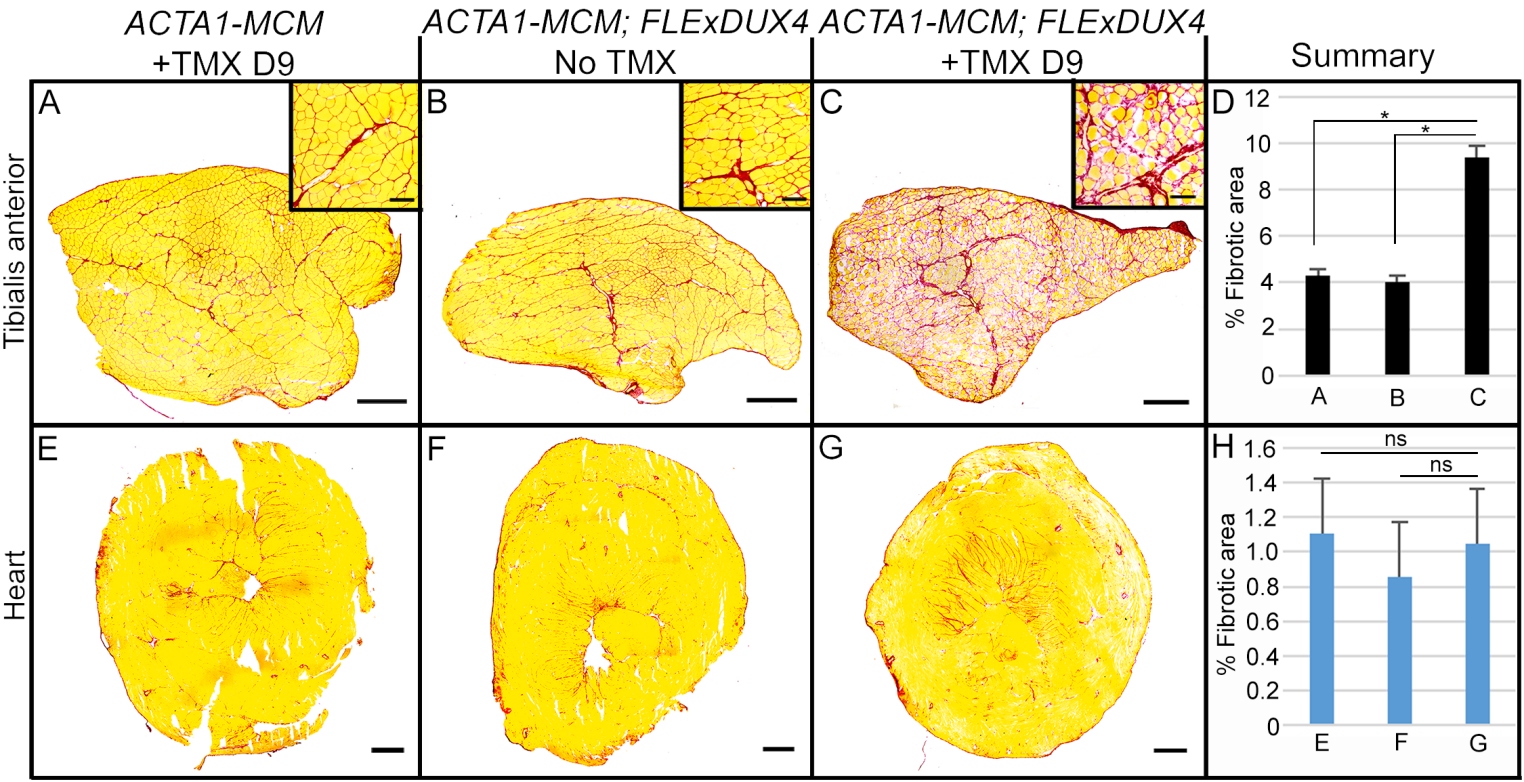
Induction of DUX4-FL expression leads to fibrosis in skeletal muscles of *ACTA1-MCM;FLExDUX4* mice. Picrosirius red staining on tibialis anterior (A-C) and heart (E-G), and summarized in (D and H), shows significantly increased fibrosis in skeletal muscles after 9 days of TMX treatment but not in the hearts of *ACTA1-MCM;FLExDUX4* mice. (D and H) Combined results from muscle obtained from 3 mice per group, 5 non-consecutive cross-section sections analyzed per muscle. Significance (* p< 0.01) was calculated using Welch’s t-test; n.s. = not significant. Bars = 500μm and 100 μm (insets).

All mice, including age-matched controls, were sacrificed nine days post-injection (13 weeks old) and muscle samples were assayed for *DUX4-fl* expression and histopathology. Surprisingly, despite the clear phenotypes, significant increases in the steady state levels of *DUX4-fl* mRNA were not detected by qRT-PCR in the phenotypic *ACTA1-MCM;FLExDUX4* (+TMX) mice (Fig 9A). However, immunostaining revealed mosaic DUX4-FL protein expression in the myonuclei (Fig 10I–10L), reminiscent of the sporadic natural bursting of expression seen in FSHD myonuclei, and three downstream target genes, *Wfdc3*, *Trim36*, and *Cxcr4* (Fig 9B–9D), were greatly induced by TMX in the skeletal muscles of *ACTA1-MCM;FLExDUX4* mice. Thus, detection of these DUX4-FL downstream targets is a more accurate method for assessing DUX4-FL levels than direct detection of *DUX4-fl* mRNA by qRT-PCR. In addition, skeletal muscle tissues that expressed DUX4-FL showed large infiltrates of mononuclear cells (Figs 10I and 11L and S12 Fig) and became mildly fibrotic (Fig 12). Thus, initial analysis of the skeletal muscles of phenotypic *ACTA1-MCM;FLExDUX4* mice indicate they share three key pathological characteristics of FSHD muscle: mosaic DUX4-FL expression that leads to DUX4-FL target gene expression, mononuclear cell infiltration, and increased fibrosis [5, 14, 15, 21, 61, 66].

Histological analysis of the tibialis anterior, gastrocnemius, and quadriceps muscles (Fig 11) showed severe pathology in the TMX-induced double transgenic animals, including the highly heterogeneous fiber size distribution characteristic of regeneration, mononuclear cell infiltration, and a staining pattern indicative of necrosis and phagocytosis (Fig 11I–11L). Similarly, the tibialis anterior muscles in the TMX-induced double transgenic animals showed increased fibrosis (Fig 12A–12D), indicative of aberrant deposition of extra-cellular matrix components during tissue healing, resulting in the loss of muscle architecture common in many muscular dystrophies including FSHD [66–69]. These phenotypes were absent from the muscles of the double transgenic mice without TMX (Fig 11E–11H) and *ACTA1-MCM* (Fig 11A–11D) control mice. Interestingly, while not a dramatic phenotype, the non-induced *ACTA1-MCM;FLExDUX4* showed patches of centralized nuclei and several small irregular fibers, indicative of muscle damage and regeneration (Fig 11H) that are not found in muscles from the *FLExDUX4/+* mice. This correlated with slightly higher levels of *DUX4-fl* mRNA and target gene expression than those found in skeletal muscle from single transgenic mice (Fig 9), due to a low level of leaky nuclear MerCreMer specifically in skeletal muscle leading to very low mosaic recombination of the transgene (S5 Fig). Thus, these non-induced double transgenic mice alone may be a useful model of very mild DUX4-mediated pathology.

**Fig 13:**
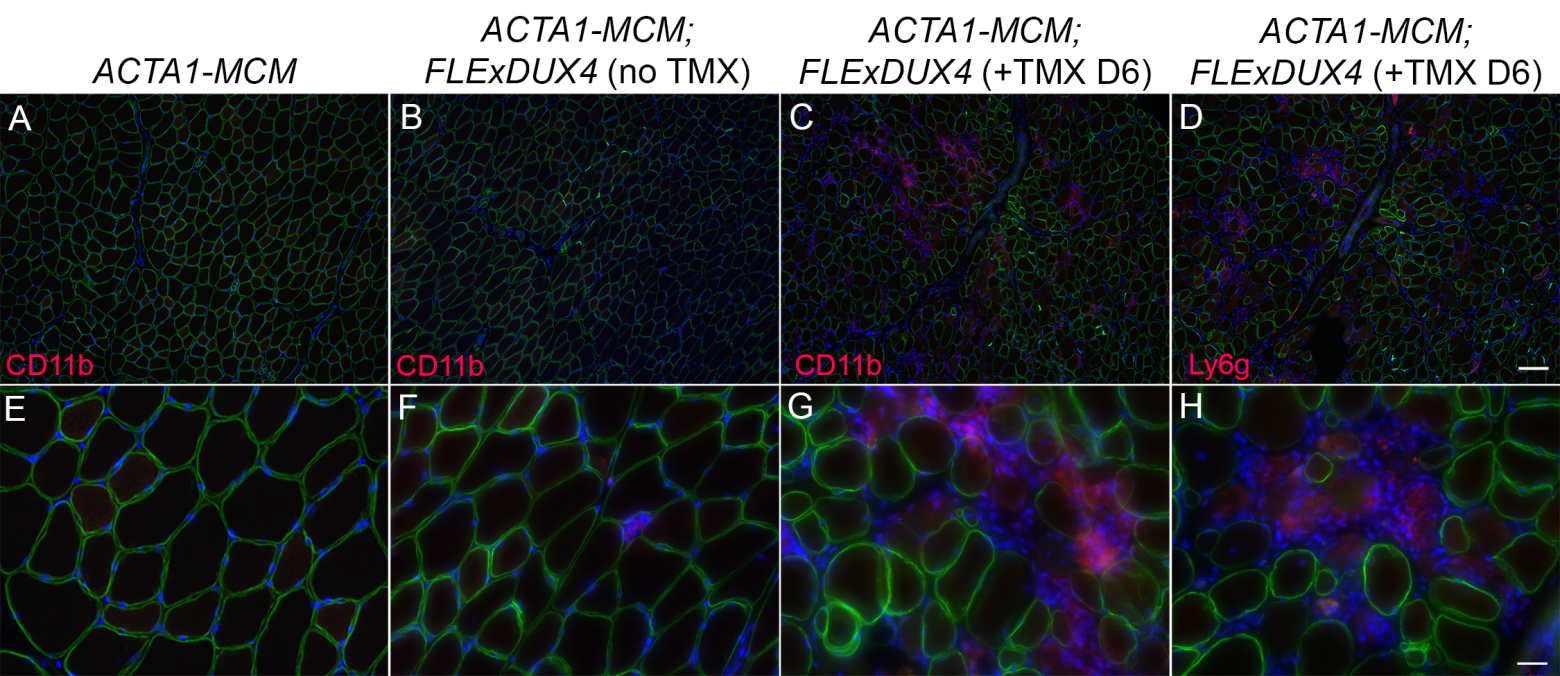
Induction of DUX4-fl leads to innate immune cell infiltration in skeletal muscles of *ACTA1-MCM;FLExDUX4* mice. Gastrocnemius muscle sections from (A, E) ACTA1-MCM, (B-D and F-H) ACTA1-MCM;FLExDUX4 mice immunostained for CD11b (A-C, and E-G) or Ly6g (E and H) reveal immune cell infiltration upon TMX induction of DUX4-fl expression. Panels (A-D) 10X magnification, Scale bar = 100μm, (E-H) 40X magnification, Scale bar = 25μm.

**Fig 14:**
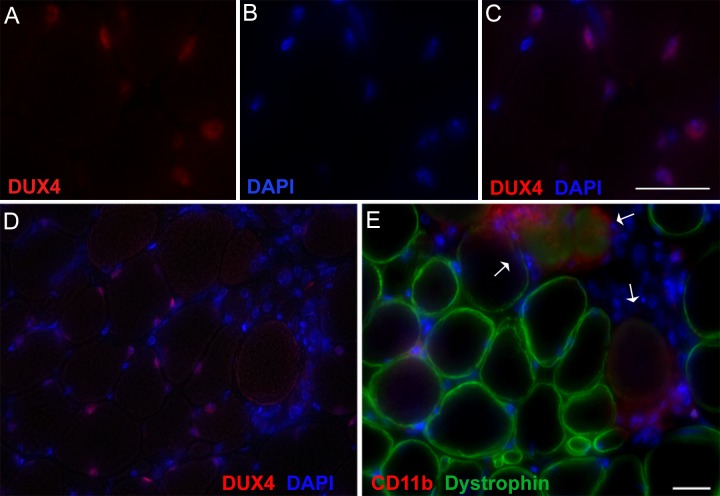
Induction of DUX4-fl leads to accumulation of immune cells, loss of membrane integrity, and a necrotic myopathy in skeletal muscles of *ACTA1-MCM;FLExDUX4* mice. (A-C) Tibialis anterior muscles from myopathic *ACTA1-MCM;FLExDUX4* mice 8 days post TMX treatment exhibit mosaic nuclear expression of DUX4-FL and (D, E) accumulation of CD11b+ cells around damaged and dying myofibers, distinguished by a loss of dystrophin localization (arrows). Scale bars = 25μm.

FSHD, like many myopathies, elicits an inflammatory immune response in affected muscles that, in turn, may contribute to muscle pathophysiology [15, 16, 61, 70, 71]. Investigation of the mononuclear cell infiltrates observed in the *ACTA1-MCM;FLExDUX4* skeletal muscle histograms by immunostaining for CD11b (*Itgam*, Integrin subunit alpha M), a member of the CD18 integrin family of leukocyte adhesion receptors involved in leukocyte migration [72], and Ly6g (lymphocyte antigen 6 complex, locus (G), a marker for murine neutrophils [73, 74], confirmed the presence of pro-inflammatory cells in the affected muscle within six days of *DUX4-fl* induction (Fig 13C, 13D, 13G and 13H). These immune cell infiltrates were absent from healthy control muscles (Fig 13A, 13B, 13E, 13F and S13 Fig). By nine days, muscles were severely affected, with massive immune cell infiltration and loss of membrane integrity, as indicated by loss of dystrophin localization (Fig 14 and S12 Fig). Importantly, these immune phenotypes are consistent with FSHD pathology as identified by MRI and histopathology; thus, this model may be useful for characterizing the role of the immune system in FSHD.

## Discussion

FSHD is a dominant genetic disease with a critical epigenetic component resulting in mis-expression of the cytotoxic isoform of the old-world primate *DUX4* gene [10]. Aberrant myogenic expression of DUX4-fl is considered to be the main driver of pathology in FSHD. However, due to the extreme cytotoxicity of DUX4-fl, FSHD has been very difficult to model in mice [23, 24, 75]. Here we report the generation and characterization of a conditional DUX4-fl transgenic mouse model, termed *FLExDUX4*. Crossing *FLExDUX4* mice with cre driver lines of mice leads to increased *DUX4-fl* mRNA and protein expression. Use of muscle-specific cre-driver lines of mice results in double transgenic offspring that express *DUX4-fl* mRNA in skeletal muscles and develop an FSHD-like myopathic phenotype [4]. Thus, these mice should be of great use for investigating pathogenic mechanisms and testing therapeutic approaches for FSHD.

Key features of the *FLExDUX4* mouse model include the following: 1) the *DUX4* transgene maintains the 3 exon, 2 intron gene structure, including the *DUX4-fl* 5’ UTR, endogenous PAS, and other important mRNA and RNA processing therapeutic targets, 2) the *DUX4-fl* mRNA expressed in skeletal muscle is spliced and polyadenylated as in FSHD patients, 3) hemizygous and homozygous *FLExDUX4* single transgenic mice are healthy, fertile, and have normal life spans, 4) *FLExDUX4* single transgenic mice express a very low level of *DUX4* RNA in all tissues tested, and *DUX4-fl* mRNA in skeletal muscles, and 5) crossing with any number of cre-driver lines will allow investigators spatiotemporal control of *DUX4-fl* expression during development or in adults.

When *FLExDUX4* mice are crossed with cre-expressing lines of mice, severe phenotypes can be obtained. Crossing *FLExDUX4* mice with *ACTA1-MCM* mice [54], a skeletal muscle-specific, TMX-inducible cre-expressing strain, produces double transgenic offspring that themselves represent a mild model of FSHD, and can be induced to develop a severe DUX4-dependent FSHD-like myopathy with low levels of TMX injected IP into adult animals. Upon TMX induction, *ACTA1-MCM;FLExDUX4* mice exhibit decreased movement, an altered gait, and loss of hanging ability (S3–S10 Movies), indicating an overall decline in muscle function. Analysis of the skeletal muscles of *ACTA1-MCM;FLExDUX4* (+TMX) mice identified other important features characteristic of FSHD. In FSHD, *DUX4-fl* mRNA is expressed in a small fraction of myonuclei in skeletal muscles, and in myogenic cells in culture [5]. Similarly, our severe FSHD-like model shows mosaic DUX4-FL protein expression in myonuclei within several days of TMX administration (Figs 10 and 14 and S12 Fig). In humans, DUX4-FL is a transcriptional activator, and induction of DUX4-FL target genes is a signature of FSHD muscle [20]. Since DUX4-fl mRNA and protein levels are typically very low in FSHD myogenic cells, DUX4 target genes serve as sensitive surrogate markers for levels of DUX4-FL expression. While DUX4-fl mRNA and protein levels are also very low in this model, the mRNA levels of DUX4-responsive genes (*Wfdc3*, *Trim36*, and *Cxcr4*) are significantly increased following DUX4-fl induction (Fig 9). Although the murine and human DUX4-induced transcriptomes only partially overlap, measuring the expression of these murine DUX4 target genes will be useful for evaluating DUX4-fl levels during therapeutic interventions in this mouse model.

FSHD muscle biopsies and MRI data indicate an inflammatory immune response in muscles of many FSHD patients [15, 16, 61, 70] and histopathology shows fibro-fatty replacement in affected FSHD muscle [66]. Inflammatory cells are able to stimulate fibrotic activity during regeneration and promote fibrotic tissue remodeling in Duchenne muscular dystrophy [76, 77]. Similarly, in the dystrophic *ACTA1-MCM;FLExDUX4* TMX-induced mice, mosaic myogenic expression of DUX4-fl leads to an infiltration of inflammatory mononuclear cells, including neutrophils and macrophages (Figs 11, 13 and 14 and S12 Fig), and increased fibrosis (Fig 12). Thus, these FSHD-like mice may represent a useful model for investigating the role of the immune response in DUX4-mediated pathophysiology, including fibrosis, and for testing therapies targeting the immune system, such as anti-inflammatories.

## Conclusions

This study represents the generation and initial characterization of the conditional cre-inducible *FLExDUX4* line of mice to provide a foundation of information for investigators to further develop and utilize according to their individual needs. Full characterization of this model in respect to FSHD, including a global analysis of gene expression, muscle physiology, detailed muscle pathology, etc., is in progress and will be part of subsequent studies. However, since we made these mice freely available to the research community through Jackson Labs prior to any publication, and to prevent the wasting of time and resources in experimental design, it is important to disseminate relevant data on this model in a timely manner to researchers already using these mice or those interested in obtaining the mice prior to a full characterization. We show conclusively that *FLExDUX4* mice are a highly versatile model for manipulating *DUX4-fl* expression levels *in vivo*. These mice can be used alone as a model of very low-level *DUX4-fl* mRNA expression in all myocytes without the development of adverse phenotypes, or, alternatively, in combination with cre-driver lines to force higher, pathogenic levels of *DUX4-fl* expression, as desired. Importantly, we characterized *ACTA1-MCM;FLExDUX4* mice, with and without TMX, as valuable new tools for FSHD research and for pre-clinical testing of potential FSHD therapeutics targeting DUX4-fl mRNA, protein, and certain downstream effects. With the *FLExDUX4* mice already available from Jackson Labs, this initial analysis provides the platform for allowing the field to move forward with investigations of FSHD pathogenic mechanisms, and testing the efficacy of numerous potential therapeutic approaches targeting DUX4-fl mRNA and protein.

## Supporting information

S1 Sequence DUX4 transgene sequence. (DOCX)

S2 Sequence FLExDUX4 mRNA sequence. (DOCX)

S1 Fig Silent DUX4 mutations eliminate DUX4-s mRNA while maintaining DUX4-fl mRNA. (PDF)

S2 Fig DNA methylation analysis of the *DUX4* transgene shows no changes across generations. (PDF)

S3 Fig Female adult *FLExDUX4/+* mice exhibit a more severe alopecia than males. (PDF)

S4 Fig Homozygous *FLExDUX4/FLExDUX4* mice have more severe phenotypes than hemizygous *FLExDUX4/+* mice. (PDF)

S5 Fig The *DUX4* transgene does not aberrantly recombine in *FLExDUX4* mice in the absence of cre. (PDF)

S6 Fig The low levels of *DUX4-fl* mRNA expression in *FLExDUX4/+* mouse muscle do not come from bursting nuclei. (PDF)

S7 Fig Expression of *Wfdc3*, a murine DUX4-FL target gene, is not significantly induced in *FLExDUX4* hemi- and homozygous mice. (PDF)

S8 Fig Skeletal muscles of FLExDUX4 mice have normal histology. (PDF)

S9 Fig *UBC-creERT2;FLExDUX4* mice undergo low levels of transgene recombination in the absence of TMX. (PDF)

S10 Fig *DUX4-fl* expression leads to enlarged lymph nodes, suggesting a DUX4-mediated immune response. (PDF)

S11 Fig TMX induction of DUX4-fl leads to physical decline and muscle loss in *ACTA1-MCM*, *FLExDUX4* mice. (PDF)

S12 Fig TMX induces DUX4-FL protein and mononuclear cell infiltration in *ACTA1-MCM;FLExDUX4* double transgenic mice. (PDF)

S13 Fig Neutrophils do not accumulate in muscles of control mice. (PDF)

S1 Movie *UBC-creERT2;FLExDUX4* (12 wks) have kyphosis and difficulty with balance. (MP4)

S2 Movie *UBC-creERT2;FLExDUX4* mice (12wks) exhibit wild running. (MP4)

S3 Movie *ACTA1-MCM;FLExDUX4*, *ACTA1-MCM/+*, and *FLExDUX4/+* mice 8 days after exposure to TMX chow and only the *ACTA1-MCM;FLExDUX4* mice exhibit difficulty moving around the cage. (MP4)

S4 Movie *FLExDUX4/+* (mouse 7b, +TMX IP injection) shows normal movement. (MP4)

S5 Movie *ACTA1-MCM*;*FLExDUX4* (mouse 6b, No TMX) shows normal movement. (MP4)

S6 Movie *ACTA1-MCM*;*FLExDUX4* (mouse 3b, +TMX IP injection) displays impaired movement. (MP4)

S7 Movie *FLExDUX4/+* (mouse 7b, +TMX IP injection) hanging test for full 2 minutes. (MP4)

S8 Movie *ACTA1-MCM*;*FLExDUX4* (mouse 6b, No TMX) hanging test for full 2 minutes. (MP4)

S9 Movie *ACTA1-MCM*;*FLExDUX4* (mouse 3b, +TMX IP injection) hanging test <2 seconds. (MP4)

S10 Movie *ACTA1-MCM*;*FLExDUX4* (mouse 4b, +TMX IP injection) hanging test <2 seconds. (MP4)
