# Supplementary material for: A cre-inducible DUX4 transgenic mouse model for investigating facioscapulohumeral muscular dystrophy
Source: PLoS One. 2018 Feb 7;13(2):e0192657. doi: 10.1371/journal.pone.0192657 (PMC5802938; doi:10.1371/journal.pone.0192657)
Supplement: S1 Sequence — (DOCX) [file pone.0192657.s001.docx]

LOCUS FLExDUX4_transge 2691 bp ds-DNA linear 14-FEB-2017

FEATURES Location/Qualifiers

misc_feature 1..27

/label=5' UTR

/ApEinfo_fwdcolor="#fff90e"

/ApEinfo_revcolor="green"

/ApEinfo_graphicformat="arrow_data {{0 1 2 0 0 -1} {} 0}

width 5 offset 0"

misc_feature 28..30

/label=ATG for DUX4-fl

/ApEinfo_fwdcolor="#13ff52"

/ApEinfo_revcolor="green"

/ApEinfo_graphicformat="arrow_data {{0 1 2 0 0 -1} {} 0}

width 5 offset 0"

misc_feature 28..1299

/label=DUX4-fl

/ApEinfo_fwdcolor="#ff70f7"

/ApEinfo_revcolor="#ff70f7"

/ApEinfo_graphicformat="arrow_data {{0 1 2 0 0 -1} {} 0}

width 5 offset 0"

misc_feature 1300..1302

/label=Stop codon

/ApEinfo_fwdcolor="#ff1f2b"

/ApEinfo_revcolor="green"

/ApEinfo_graphicformat="arrow_data {{0 1 2 0 0 -1} {} 0}

width 5 offset 0"

misc_feature 1311..1446

/label=Intron 1

/ApEinfo_fwdcolor="cyan"

/ApEinfo_revcolor="cyan"

/ApEinfo_graphicformat="arrow_data {{0 1 2 0 0 -1} {} 0}

width 5 offset 0"

misc_feature 1447..1536

/label=Exon 2

/ApEinfo_fwdcolor="#ff89e0"

/ApEinfo_revcolor="#ff89e0"

/ApEinfo_graphicformat="arrow_data {{0 1 2 0 0 -1} {} 0}

width 5 offset 0"

misc_feature 1537..1898

/label=Intron 2

/ApEinfo_fwdcolor="cyan"

/ApEinfo_revcolor="cyan"

/ApEinfo_graphicformat="arrow_data {{0 1 2 0 0 -1} {} 0}

width 5 offset 0"

misc_feature 1899..2067

/label=Exon 3

/ApEinfo_fwdcolor="#d94aff"

/ApEinfo_revcolor="green"

/ApEinfo_graphicformat="arrow_data {{0 1 2 0 0 -1} {} 0}

width 5 offset 0"

misc_feature 2068..2099

/label=Exon 3(1)

/ApEinfo_label="Exon 3"

/ApEinfo_fwdcolor="#d94aff"

/ApEinfo_revcolor="green"

/ApEinfo_graphicformat="arrow_data {{0 1 2 0 0 -1} {} 0}

width 5 offset 0"

misc_feature 2068..2073

/label=Poly A site

/ApEinfo_fwdcolor="#baff7b"

/ApEinfo_revcolor="green"

/ApEinfo_graphicformat="arrow_data {{0 1 2 0 0 -1} {} 0}

width 5 offset 0"

misc_feature 2100..2308

/label=pLam Region

/ApEinfo_fwdcolor="#ff790b"

/ApEinfo_revcolor="green"

/ApEinfo_graphicformat="arrow_data {{0 1 2 0 0 -1} {} 0}

width 5 offset 0"

misc_feature 2328..2361

/label=lox511

/ApEinfo_fwdcolor="#11ff33"

/ApEinfo_revcolor="#11ff33"

/ApEinfo_graphicformat="arrow_data {{0 1 2 0 0 -1} {} 0}

width 5 offset 0"

misc_feature complement(2664..2691)

/label=TJ76F (105116flp-PJ02)

/ApEinfo_fwdcolor="#ff291d"

/ApEinfo_revcolor="#ff291d"

/ApEinfo_graphicformat="arrow_data {{0 1 2 0 0 -1} {} 0}

width 5 offset 0"

misc_recomb 2376..2409

/label=FRT

/ApEinfo_fwdcolor="#dab3a9"

/ApEinfo_revcolor="#dab3a9"

/ApEinfo_graphicformat="arrow_data {{0 1 2 0 0 -1} {} 0}

width 5 offset 0"

misc_recomb 2410..2443

/label=LoxP

/ApEinfo_fwdcolor="#82aaff"

/ApEinfo_revcolor="#82aaff"

/ApEinfo_graphicformat="arrow_data {{0 1 2 0 0 -1} {} 0}

width 5 offset 0"

ORIGIN

1 GTGAAATTCC GGCCGGGGCT CACCGCGATG GCCCTCCCGA CACCCTCGGA CAGCACCCTC

61 CCCGCGGAAG CCCGGGGACG AGGACGGCGA CGGAGACTCG TTTGGACCCC GAGCCAAAGC

121 GAGGCCCTGC GAGCCTGCTT TGAGCGGAAC CCGTACCCGG GCATCGCCAC CAGAGAACGG

181 CTGGCCCAGG CCATCGGCAT TCCGGAGCCC AGGGTCCAGA TTTGGTTTCA GAATGAGAGG

241 TCACGCCAGC TGAGGCAGCA CCGGCGGGAA TCTCGGCCCT GGCCCGGGAG ACGCGGCCCG

301 CCAGAAGGCC GGCGAAAGCG GACCGCCGTC ACCGGATCCC AGACCGCCCT GCTCCTCCGA

361 GCCTTTGAGA AGGATCGCTT TCCGGGGATT GCTGCCCGGG AGGAGCTGGC CAGAGAGACG

421 GGCCTCCCGG AGTCCAGGAT TCAGATCTGG TTTCAGAATC GAAGGGCCAG GCACCCGGGA

481 CAGGGTGGCA GGGCGCCCGC GCAAGCCGGT GGCCTGTGCA GCGCGGCCCC CGGCGGGGGT

541 CACCCTGCTC CCTCGTGGGT CGCCTTCGCC CACACCGGCG CGTGGGGAAC GGGGCTTCCC

601 GCACCCCACG TGCCCTGCGC GCCTGGGGCT CTCCCACAGG GGGCTTTCGT GAGCCAGGCA

661 GCGAGGGCCG CCCCCGCGCT GCAGCCCAGC CAGGCCGCGC CGGCAGAGGG GATCTCCCAA

721 CCTGCCCCGG CGCGCGGGGA TTTCGCCTAC GCCGCCCCGG CTCCTCCGGA CGGGGCGCTC

781 TCCCACCCTC AGGCTCCTCG CTGGCCTCCG CACCCGGGCA AAAGCCGGGA GGACCGGGAC

841 CCGCAGCGCG ACGGCCTGCC GGGCCCCTGC GCGGTGGCAC AGCCTGGGCC CGCTCAAGCG

901 GGGCCGCAGG GCCAAGGGGT GCTTGCGCCA CCCACGTCCC AGGGGAGTCC GTGGTGGGGC

961 TGGGGCCGGG GTCCCCAGGT CGCCGGGGCG GCGTGGGAAC CCCAAGCCGG GGCAGCTCCA

1021 CCTCCCCAGC CCGCGCCCCC GGACGCCTCC GCCTCCGCGC GGCAGGGGCA GATGCAAGGC

1081 ATCCCGGCGC CCTCCCAGGC GCTCCAGGAG CCGGCGCCCT GGTCTGCACT CCCCTGCGGC

1141 CTGCTGCTGG ATGAGCTCCT GGCGAGCCCG GAGTTTCTGC AGCAGGCGCA ACCTCTCCTA

1201 GAAACGGAGG CCCCGGGGGA GCTGGAGGCC TCGGAAGAGG CCGCCTCGCT GGAAGCACCC

1261 CTCAGCGAGG AAGAATACCG GGCTCTGCTG GAGGAGCTTT AGGACGCGGG GTTGGGACGG

1321 GGTCGGGTGG TTCGGGGCAG GGCGGTGGCC TCTCTTTCGC GGGGAACACC TGGCTGGCTA

1381 CGGAGGGGCG TGTCTCCGCC CCGCCCCCTC CACCGGGCTG ACCGGCCTGG GATTCCTGCC

1441 TTCTAGGTCT AGGCCCGGTG AGAGACTCCA CACCGCGGAG AACTGCCATT CTTTCCTGGG

1501 CATCCCGGGG ATCCCAGAGC CGGCCCAGGT ACCAGCAGGT GGGCCGCCTA CTGCGCACGC

1561 GCGGGTTTGC GGGCAGCCGC CTGGGCTGTG GGAGCAGCCC GGGCAGAGCT CTCCTGCCTC

1621 TCCACCAGCC CACCCCGCCG CCTGACCGCC CCCTCCCCAC CCCCACCCCC CACCCCCGGA

1681 AAACGCGTCG TCCCCTGGGC TGGGTGGAGA CCCCCGTCCC GCGAAACACC GGGCCCCGCG

1741 CAGCGTCCGG GCCTGACACC GCTCCGGCGG CTCGCCTCCT CTGCGCCCCC GCGCCACCGT

1801 CGCCCGCCCG CCCGGGCCCC TGCAGCCTCC CAGCTGCCAG CGCGGAGCTC CTGGCGGTCA

1861 AAAGCATACC TCTGTCTGTC TTTGCCCGCT TCCTGACTAG ACCTGCGCGC AGTGCGCACC

1921 CCGGCTGACG TGCAAGGGAG CTCGCTGGCC TCTCTGTGCC CTTGTTCTTC CGTGAAATTC

1981 TGGCTGAATG TCTCCCCCCA CCTTCCGACG CTGTCTAGGC AAACCTGGAT TAGAGTTACA

2041 TCTCCTGGAT GATTAGTTCA GAGATATATT AAAATGCCCC CTCCCTGTGG ATCCTATAGA

2101 AGATTTGCAT CTTTTGTGTG ATGAGTGCAG AGATATGTCA CAATATCCCC TGTAGAAAAA

2161 GCCTGAAATT GGTTTACATA ACTTCGGTGA TCAGTGCAGA TGTGTTTCAG AACTCCATAG

2221 TAGACTGAAC CTAGAGAATG GTTACATCAC TTAGGTGATC AGTGTAGAGA TATGTTAAAA

2281 TTCTCGTGTA GACAGAGCCT AGACAATTTG TTGGCGCGTC ATTTGCTata acttcgtata

2341 ATGTATACta tacgaagtta tCCTAGGGCG GCCGCGAAGT TCCTATTCTC TAGAAAGTAT

2401 AGGAACTTCA TAACTTCGTA TAATGTATGC TATACGAAGT TATATTTAAA TCGCCCGGGG

2461 TTAACTACGT GTTGGGCCAA AGAGAGATGC AGTGAGAAGA GTACCACCAA GAGTCCAATG

2521 ATTGCACCGA AGAGTTTGTC CTCAACCGCG AGCTGTGGAA AAAAAAGGGA TTAATTAAGG

2581 CTAGAAAGAC TGGAGTTGCA GATCACGAGG GAAGAGGGGG AAGGGATTCT CCCAGGCCCA

2641 GGGCGGTCCT CAGAAGCCAG GAGGCAGCAG AGAACTCCCA GAAAGGTATT G

//
