# Supplementary material for: A cre-inducible DUX4 transgenic mouse model for investigating facioscapulohumeral muscular dystrophy
Source: PLoS One. 2018 Feb 7;13(2):e0192657. doi: 10.1371/journal.pone.0192657 (PMC5802938; doi:10.1371/journal.pone.0192657)
Supplement: S2 Sequence — (DOCX) [file pone.0192657.s002.docx]

LOCUS FLExD_mRNA_ 1711 bp ds-DNA linear 31-JUL-2017

DEFINITION .

ACCESSION

VERSION

SOURCE .

ORGANISM .

COMMENT

COMMENT ApEinfo:methylated:0

FEATURES Location/Qualifiers

misc_feature 1300..1302

/label=Stop codon

/ApEinfo_fwdcolor="#ff1f2b"

/ApEinfo_revcolor="green"

/ApEinfo_graphicformat="arrow_data {{0 1 2 0 0 -1} {} 0}

width 5 offset 0"

misc_feature 1537..1705

/label=Exon 3

/ApEinfo_fwdcolor="#d94aff"

/ApEinfo_revcolor="green"

/ApEinfo_graphicformat="arrow_data {{0 1 2 0 0 -1} {} 0}

width 5 offset 0"

misc_feature 1..27

/label=5' UTR

/ApEinfo_fwdcolor="#fff90e"

/ApEinfo_revcolor="green"

/ApEinfo_graphicformat="arrow_data {{0 1 2 0 0 -1} {} 0}

width 5 offset 0"

misc_feature 1447..1536

/label=Exon 2

/ApEinfo_fwdcolor="#ff89e0"

/ApEinfo_revcolor="#ff89e0"

/ApEinfo_graphicformat="arrow_data {{0 1 2 0 0 -1} {} 0}

width 5 offset 0"

misc_feature 28..1299

/label=DUX4-fl

/ApEinfo_fwdcolor="#ff70f7"

/ApEinfo_revcolor="#ff70f7"

/ApEinfo_graphicformat="arrow_data {{0 1 2 0 0 -1} {} 0}

width 5 offset 0"

misc_feature 28..30

/label=ATG for DUX4-fl

/ApEinfo_fwdcolor="#13ff52"

/ApEinfo_revcolor="green"

/ApEinfo_graphicformat="arrow_data {{0 1 2 0 0 -1} {} 0}

width 5 offset 0"

misc_feature 1311..1446

/label=Intron 1

/ApEinfo_fwdcolor="cyan"

/ApEinfo_revcolor="cyan"

/ApEinfo_graphicformat="arrow_data {{0 1 2 0 0 -1} {} 0}

width 5 offset 0"

misc_feature 1706..1711

/label=Exon 3(1)

/ApEinfo_label="Exon 3"

/ApEinfo_fwdcolor="#d94aff"

/ApEinfo_revcolor="green"

/ApEinfo_graphicformat="arrow_data {{0 1 2 0 0 -1} {} 0}

width 5 offset 0"

misc_feature 1706..1711

/label=Poly A site

/ApEinfo_fwdcolor="#baff7b"

/ApEinfo_revcolor="green"

/ApEinfo_graphicformat="arrow_data {{0 1 2 0 0 -1} {} 0}

width 5 offset 0"

ORIGIN

1 gtgaaattcc ggccggggct caccgcgATG GCCCTCCCGA CACCCTCGGA CAGCACCCTC

61 CCCGCGGAAG CCCGGGGACG AGGACGGCGA CGGAGACTCG TTTGGACCCC GAGCCAAAGC

121 GAGGCCCTGC GAGCCTGCTT TGAGCGGAAC CCGTACCCGG GCATCGCCAC CAGAGAACGG

181 CTGGCCCAGG CCATCGGCAT TCCGGAGCCC AGGGTCCAGA TTTGGTTTCA GAATGAGAGG

241 TCACGCCAGC TGAGGCAGCA CCGGCGGGAA TCTCGGCCCT GGCCCGGGAG ACGCGGCCCG

301 CCAGAAGGCC GGCGAAAGCG GACCGCCGTC ACCGGATCCC AGACCGCCCT GCTCCTCCGA

361 GCCTTTGAGA AGGATCGCTT TCCGGGGATT GCTGCCCGGG AGGAGCTGGC CAGAGAGACG

421 GGCCTCCCGG AGTCCAGGAT TCAGATCTGG TTTCAGAATC GAAGGGCCAG GCACCCGGGA

481 CAGGGTGGCA GGGCGCCCGC GCAAGCCGGT GGCCTGTGCA GCGCGGCCCC CGGCGGGGGT

541 CACCCTGCTC CCTCGTGGGT CGCCTTCGCC CACACCGGCG CGTGGGGAAC GGGGCTTCCC

601 GCACCCCACG TGCCCTGCGC GCCTGGGGCT CTCCCACAGG GGGCTTTCGT GAGCCAGGCA

661 GCGAGGGCCG CCCCCGCGCT GCAGCCCAGC CAGGCCGCGC CGGCAGAGGG GATCTCCCAA

721 CCTGCCCCGG CGCGCGGGGA TTTCGCCTAC GCCGCCCCGG CTCCTCCGGA CGGGGCGCTC

781 TCCCACCCTC AGGCTCCTCG CTGGCCTCCG CACCCGGGCA AAAGCCGGGA GGACCGGGAC

841 CCGCAGCGCG ACGGCCTGCC GGGCCCCTGC GCGGTGGCAC AGCCTGGGCC CGCTCAAGCG

901 GGGCCGCAGG GCCAAGGGGT GCTTGCGCCA CCCACGTCCC AGGGGAGTCC GTGGTGGGGC

961 TGGGGCCGGG GTCCCCAGGT CGCCGGGGCG GCGTGGGAAC CCCAAGCCGG GGCAGCTCCA

1021 CCTCCCCAGC CCGCGCCCCC GGACGCCTCC GCCTCCGCGC GGCAGGGGCA GATGCAAGGC

1081 ATCCCGGCGC CCTCCCAGGC GCTCCAGGAG CCGGCGCCCT GGTCTGCACT CCCCTGCGGC

1141 CTGCTGCTGG ATGAGCTCCT GGCGAGCCCG GAGTTTCTGC AGCAGGCGCA ACCTCTCCTA

1201 GAAACGGAGG CCCCGGGGGA GCTGGAGGCC TCGGAAGAGG CCGCCTCGCT GGAAGCACCC

1261 CTCAGCGAGG AAGAATACCG GGCTCTGCTG GAGGAGCTTT AGGACGCGGG GTTGGGACGG

1321 GGTCGGGTGG TTCGGGGCAG GGCCGTGGCC TCTCTTTCGC GGGGAACACC TGGCTGGCTA

1381 CGGAGGGGCG TGTCTCCGCC CCGCCCCCTC CACCGGGCTG ACCGGCCTGG GATTCCTGCC

1441 TTCTAGGTCT AGGCCCGGTG AGAGACTCCA CACCGCGGAG AACTGCCATT CTTTCCTGGG

1501 CATCCCGGGG ATCCCAGAGC CGGCCCAGGT ACCAGCAGAC CTGCGCGCAG TGCGCACCCC

1561 GGCTGACGTG CAAGGGAGCT CGCTGGCCTC TCTGTGCCCT TGTTCTTCCG TGAAATTCTG

1621 GCTGAATGTC TCCCCCCACC TTCCGACgct gtctaggcaa acctggatta gagttacatc

1681 tcctggatga ttagttcaga gatatattaa a

//

Uppercase confirmed by sequencing of RT-PCR product

Lowercase indicates not confirmed by sequencing
