# Supplementary figures and images for: A cre-inducible DUX4 transgenic mouse model for investigating facioscapulohumeral muscular dystrophy
Source: PLoS One. 2018 Feb 7;13(2):e0192657. doi: 10.1371/journal.pone.0192657 (PMC5802938; doi:10.1371/journal.pone.0192657)

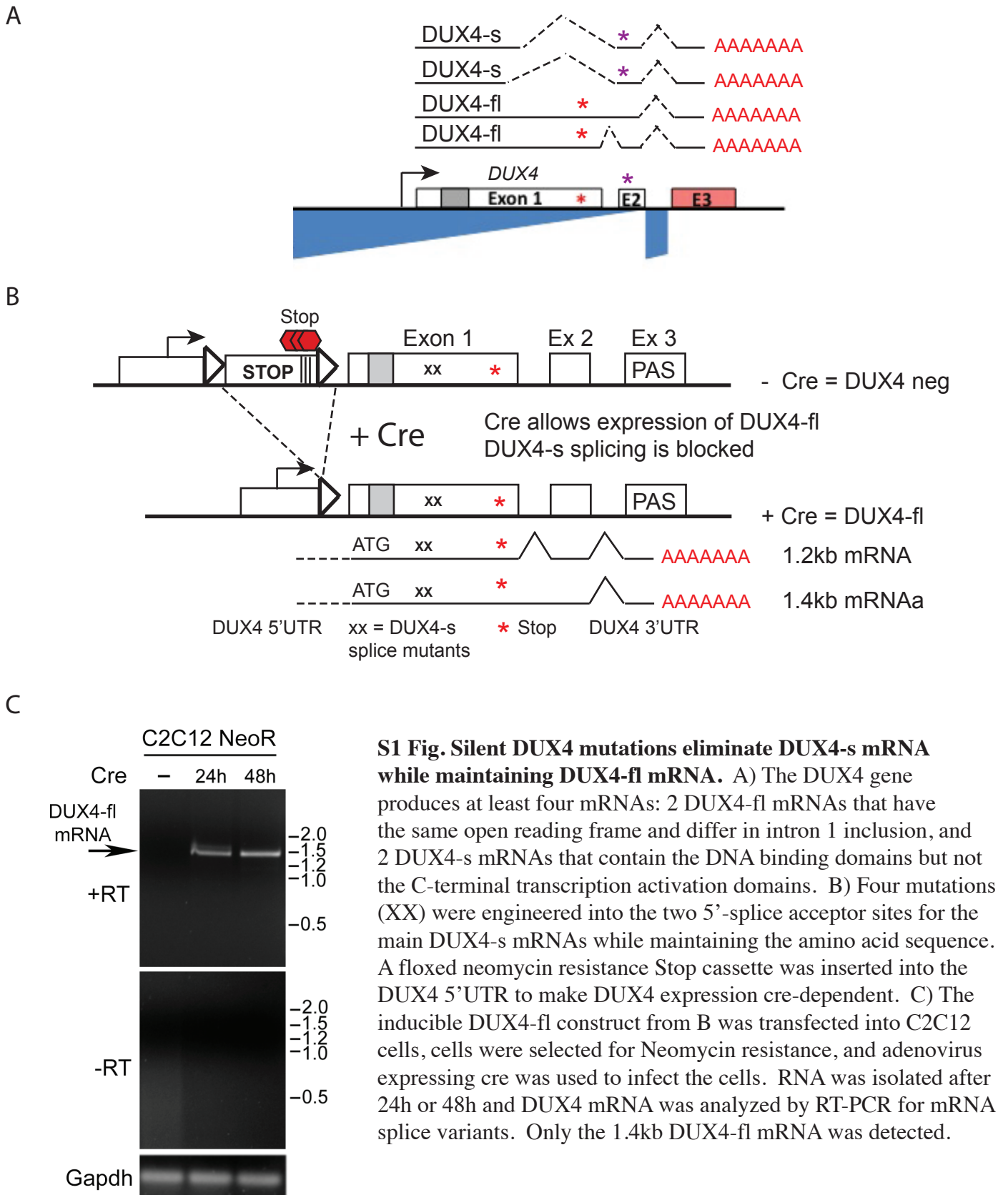

Supplement: S1 Fig — (PDF) [file pone.0192657.s003.pdf]
