# Supplementary material for: A cre-inducible DUX4 transgenic mouse model for investigating facioscapulohumeral muscular dystrophy
Source: PLoS One. 2018 Feb 7;13(2):e0192657. doi: 10.1371/journal.pone.0192657 (PMC5802938; doi:10.1371/journal.pone.0192657)
Supplement: S2 Fig — (PDF) [file pone.0192657.s004.pdf]

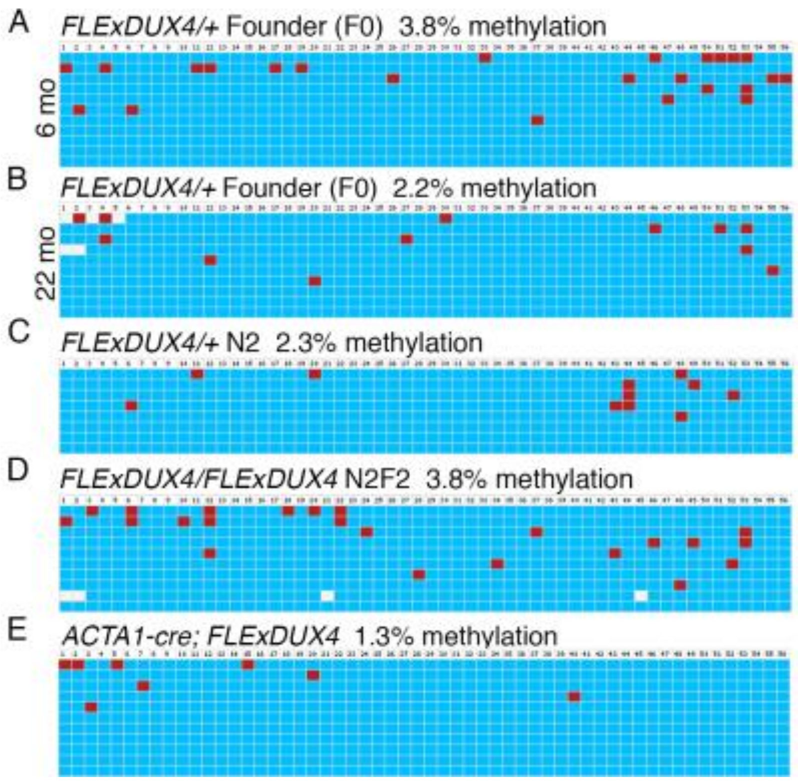

S2 Fig. DNA methylation analysis of the *DUX4* transgene shows no changes across generations. Bisulfite sequencing was performed on genomic DNA isolated from the FLExDUX4/+ founder mice at A) 6 months and B) 22 months, C) backcrossed after two generations, D) homozygous mice after 4 generations, and E) when crossed with the ACTA1-cre line. End of exon 1 and into exon 2 of the *DUX4* transgene were analyzed as described [1]. Each square indicates a predicted CpG based on the human *DUX4* sequence. Red squares indicate methylated CpGs, blue squares indicate unmethylated CpGs, and white squares indicate no predicted CpGs were detected at these positions. The transgene remained hypomethylated across ages, generations, and when crossed with a cre driver line.

1. Jones TI, Yan C, Sapp PC, McKenna-Yasek D, Kang PB, Quinn C, et al. Identifying diagnostic DNA methylation profiles for facioscapulohumeral muscular dystrophy in blood and saliva using bisulfite sequencing. Clinical epigenetics. 2014;6(1):23. doi: 10.1186/1868-7083-6-23. PubMed PMID: 25400706.
