# Supplementary material for: A cre-inducible DUX4 transgenic mouse model for investigating facioscapulohumeral muscular dystrophy
Source: PLoS One. 2018 Feb 7;13(2):e0192657. doi: 10.1371/journal.pone.0192657 (PMC5802938; doi:10.1371/journal.pone.0192657)
Supplement: S3 Fig — (PDF) [file pone.0192657.s005.pdf]

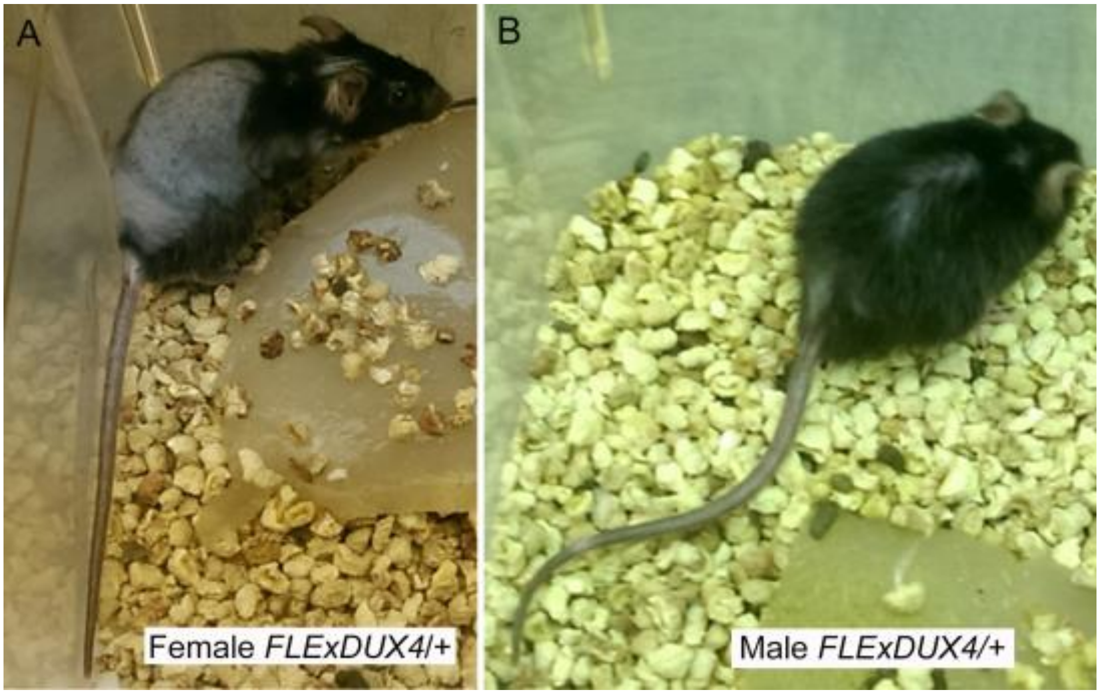

S3 Fig. Female adult *FLExDUX4/+* mice exhibit a more severe alopecia than males. In contrast to age-matched adult male *FLExDUX4/+* mice, female *FLExDUX4/+* mice are relatively healthy and of normal weight; however, they exhibit a more severe alopecia than their male counterparts.
