# Supplementary material for: A cre-inducible DUX4 transgenic mouse model for investigating facioscapulohumeral muscular dystrophy
Source: PLoS One. 2018 Feb 7;13(2):e0192657. doi: 10.1371/journal.pone.0192657 (PMC5802938; doi:10.1371/journal.pone.0192657)
Supplement: S4 Fig — (PDF) [file pone.0192657.s006.pdf]

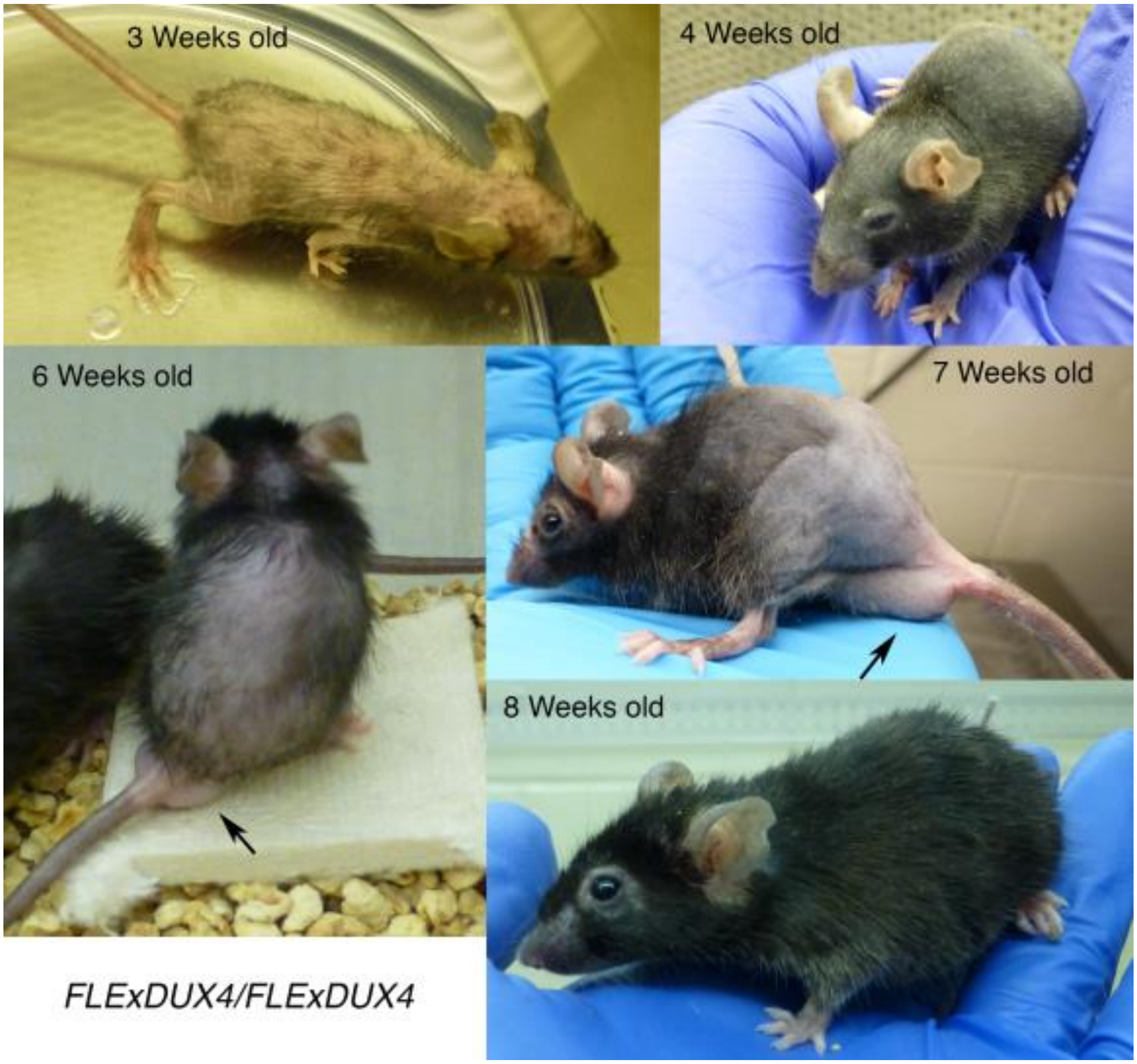

S4 Fig. Homozygous *FLExDUX4/FLExDUX4* mice have more severe phenotypes than hemizygous *FLExDUX4/+* mice. Both male (shown) and female (not shown) *FLExDUX4/FLExDUX4* mice have a characteristic severe alopecia by 3 weeks after birth that rapidly lessens with age. Adult mice regain much of their hair, but still exhibit an alopecia phenotype that is readily distinguished from hemizygous *FLExDUX4/+* mice, regardless of sex. In addition, *FLExDUX4* homozygous male mice, while very fertile, develop an inflamed preputial gland (arrow) and distended testicles (not shown).
