# Supplementary material for: A cre-inducible DUX4 transgenic mouse model for investigating facioscapulohumeral muscular dystrophy
Source: PLoS One. 2018 Feb 7;13(2):e0192657. doi: 10.1371/journal.pone.0192657 (PMC5802938; doi:10.1371/journal.pone.0192657)
Supplement: S5 Fig — (PDF) [file pone.0192657.s007.pdf]

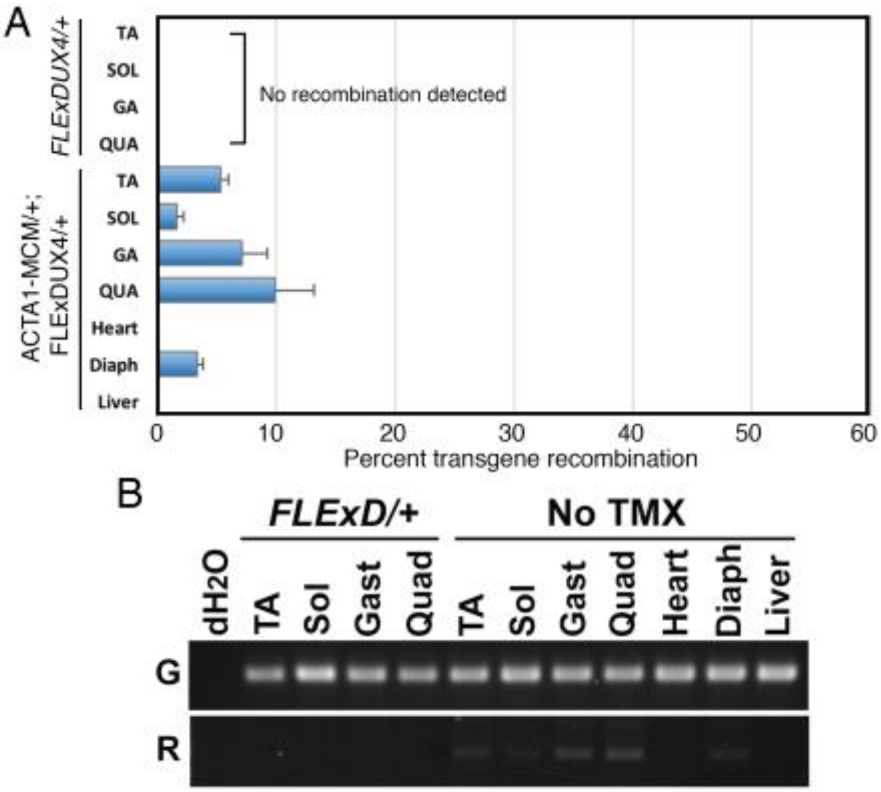

S5 Fig. The *DUX4* transgene does not aberrantly recombine in *FLExDUX4* mice in the absence of cre. Genomic DNA isolated from tissues of adult *FLExDUX4/+* and double transgenic *ACTA1-MCM/+; FLExDUX4/+* mice was subjected to PCR analysis for the (G) antisense orientation and the recombined (R) sense orientation for *DUX4-fl*. A) Quantification of recombination assayed by B) agarose gel electrophoresis of PCR products showed some recombination in *ACTA1-MCM/+; FLExDUX4/+* mice even without TMX induction of cre, however there is no detectable *DUX4* transgene recombination in the *FLExDUX4/+* single transgenic mice.
