# Supplementary material for: A cre-inducible DUX4 transgenic mouse model for investigating facioscapulohumeral muscular dystrophy
Source: PLoS One. 2018 Feb 7;13(2):e0192657. doi: 10.1371/journal.pone.0192657 (PMC5802938; doi:10.1371/journal.pone.0192657)
Supplement: S6 Fig — (PDF) [file pone.0192657.s008.pdf]

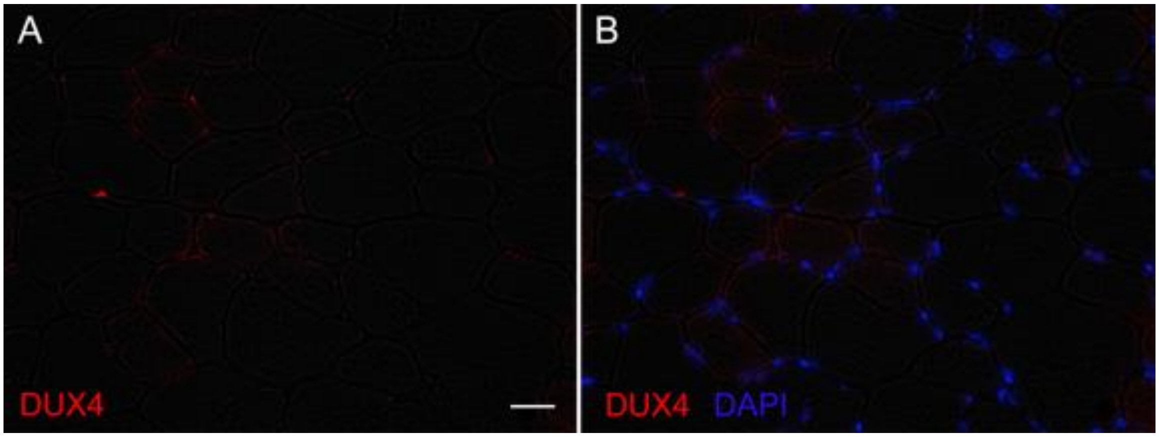

S6 Fig. The low levels of *DUX4-fl* mRNA expression in *FLExDUX4/+* mouse muscle do not come from bursting nuclei. Gastrocnemius muscle section immunostained for A) DUX4-FL protein and B) merged with DAPI staining and bright field image shows no indication of DUX4-FL positive myonuclei, consistent with the lack of detectable recombination of the transgene. Red, DUX4-FL; Blue, DAPI; Scale bar = 25µm.
