# Supplementary material for: A cre-inducible DUX4 transgenic mouse model for investigating facioscapulohumeral muscular dystrophy
Source: PLoS One. 2018 Feb 7;13(2):e0192657. doi: 10.1371/journal.pone.0192657 (PMC5802938; doi:10.1371/journal.pone.0192657)
Supplement: S7 Fig — (PDF) [file pone.0192657.s009.pdf]

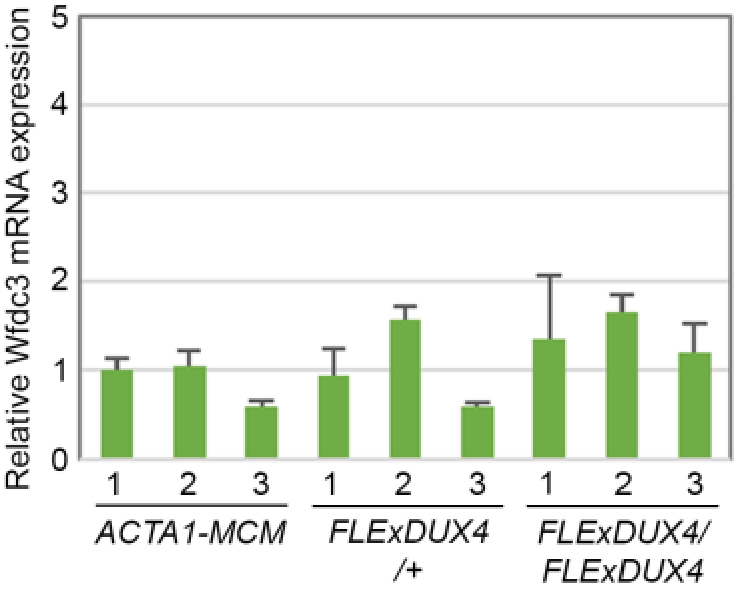

S7 Fig. Expression of *Wfdc3*, a murine DUX4-FL target gene, is not significantly induced in *FLExDUX4* hemi- and homozygous mice. Gastrocnemius muscle from 13-week-old mice was analyzed by qRT-PCR for *Wfdc3* expression, normalized to *Rpl37* expression, and plotted compared to no *DUX4-fl* control (ACTA1-MCM mouse #1 = 1.0). Results for each of three independent mice per genotype are shown.
