# Supplementary material for: A cre-inducible DUX4 transgenic mouse model for investigating facioscapulohumeral muscular dystrophy
Source: PLoS One. 2018 Feb 7;13(2):e0192657. doi: 10.1371/journal.pone.0192657 (PMC5802938; doi:10.1371/journal.pone.0192657)
Supplement: S8 Fig — (PDF) [file pone.0192657.s010.pdf]

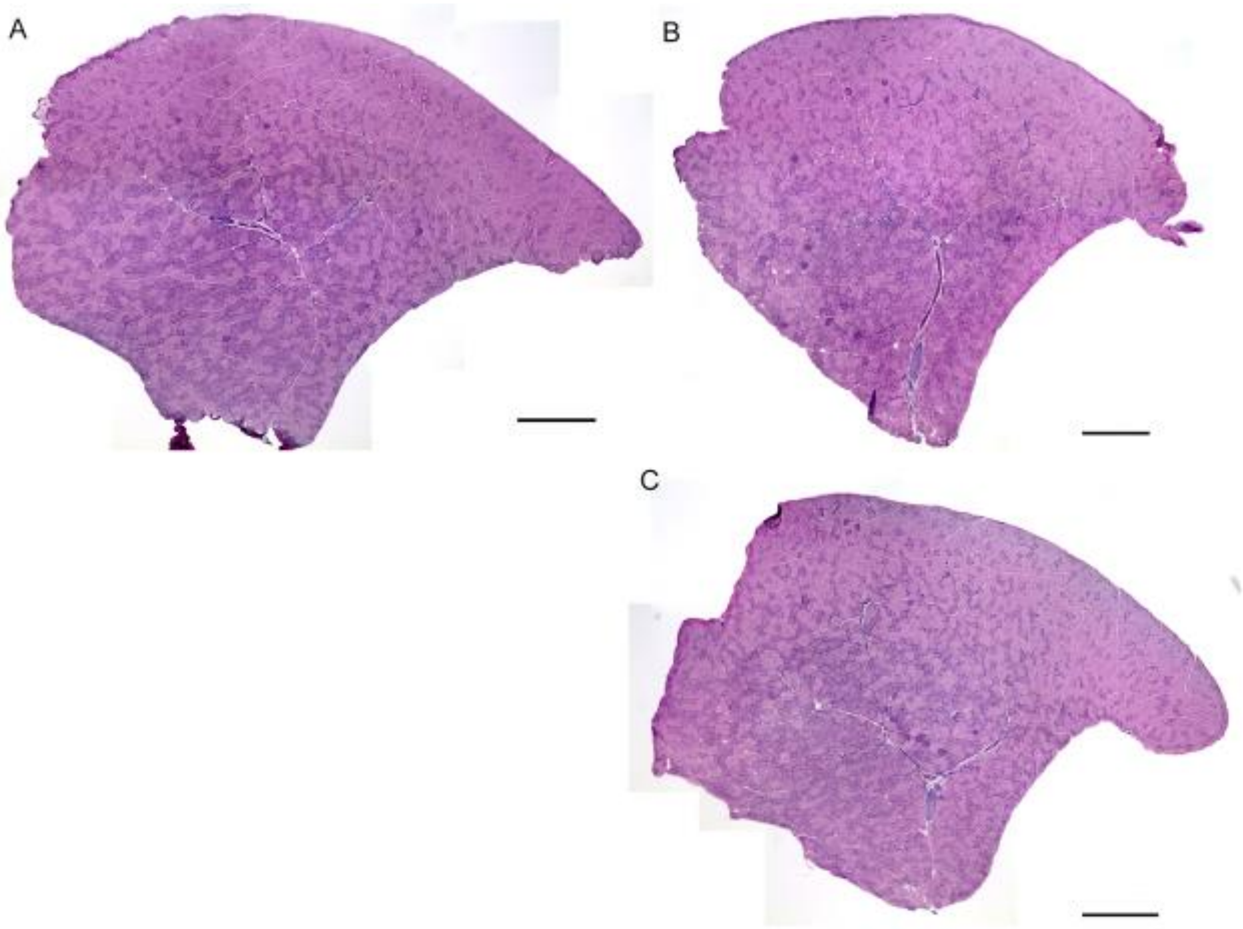

S8 Fig. Skeletal muscles of FLExDUX4 mice have normal histology. Skeletal muscles were isolated from 23-week-old female mice and histologically assessed by H&E staining. Tibialis anterior sections are shown for A) control C57BL/6, B) *FLExDUX4/+,* and C) *FLExDUX4/FLExDUX4*. Each whole cross section image was generated by combining multiple frames using Leica LAS software. Enlarged sections are found in Fig. 4. Scale bar = 500μm.
