# Supplementary material for: A cre-inducible DUX4 transgenic mouse model for investigating facioscapulohumeral muscular dystrophy
Source: PLoS One. 2018 Feb 7;13(2):e0192657. doi: 10.1371/journal.pone.0192657 (PMC5802938; doi:10.1371/journal.pone.0192657)
Supplement: S9 Fig — (PDF) [file pone.0192657.s011.pdf]

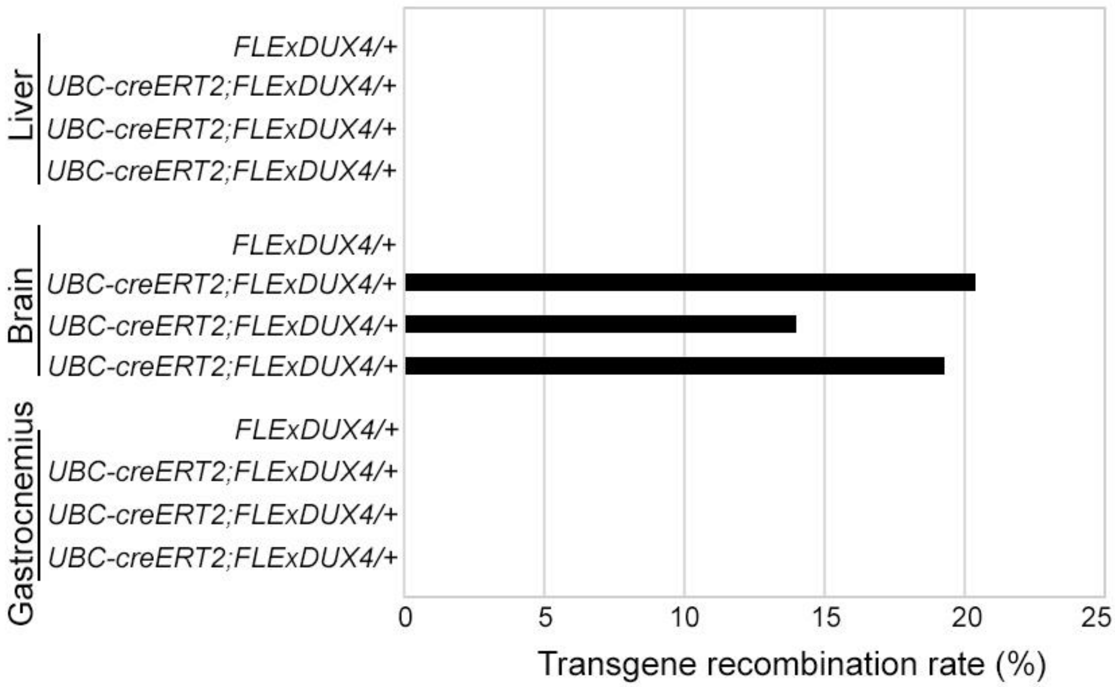

S9 Fig. *UBC-creERT2;FLExDUX4* mice undergo low levels of transgene recombination in the absence of TMX. Genomic DNA isolated from liver, brain, and gastrocnemius muscle of three *UBC-creERT2;FLExDUX4* double transgenic mice used for RNA analysis in Fig. 9 was assayed for cre-mediated recombination of the FLExDUX4 transgene.
