# Supplementary material for: A cre-inducible DUX4 transgenic mouse model for investigating facioscapulohumeral muscular dystrophy
Source: PLoS One. 2018 Feb 7;13(2):e0192657. doi: 10.1371/journal.pone.0192657 (PMC5802938; doi:10.1371/journal.pone.0192657)
Supplement: S10 Fig — (PDF) [file pone.0192657.s012.pdf]

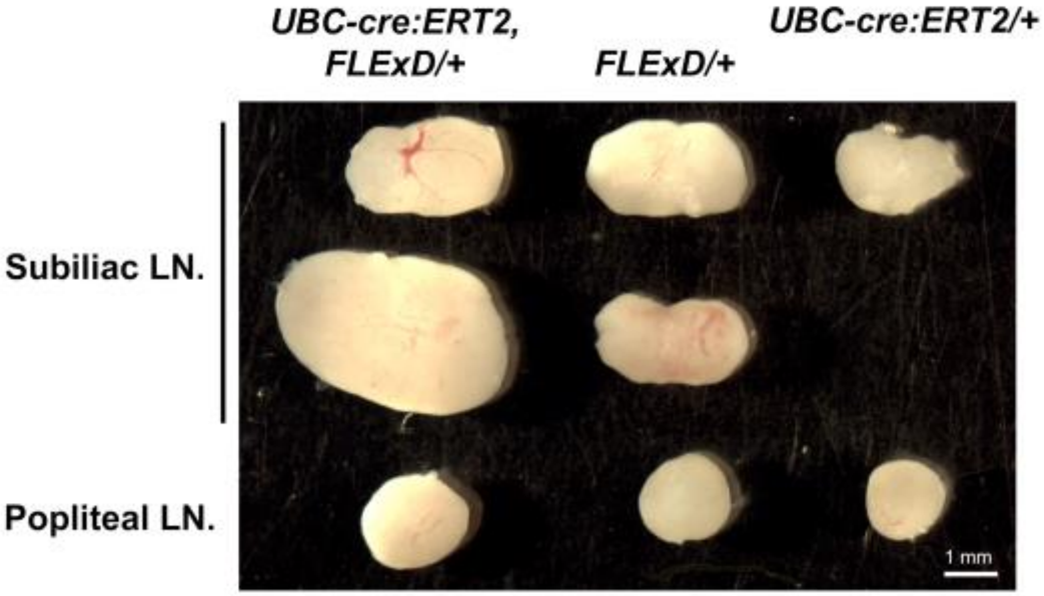

	Subiliac Lymph Nodes						Popliteal Lymph Nodes					
	Control (mg)		*FLExDUX4/+* (mg)		*UBC-creERT2; FLExDUX4* (mg)		Control (mg)		*FLExDUX4/+* (mg)		*UBC-creERT2; FLExDUX4* (mg)	
	3.0	3.0	8.0	8.0	10.0	12.0	1.0	0.9	1.8	2.0	3.6	7.5
	1.8	NF	3.0	4.5	34.0	11.0	1.1	NF	1.7	2.3	8.1	9.6
	2.8	2.1	2.7	2.7	12.8	4.1	1.4	1.0	1.0	0.9	2.6	NF
	3.0	3.2	4.1	3.4	10.6	14.0	0.9	0.7	2.2	NF	2.5	2.4
	3.1	3.5	3.3	NF	8.7	8.2	1.2	NF	1.7	1.2	1.5	1.7
	3.1	1.5	4.0	3.7			1.0	1.4	2.5	1.8		
	2.5	1.9	4.4	3.6			0.8	1.0	1.5	NF		
	1.9	2.5	3.7	4.5			0.2	0.9	1.7	1.4		
	2.2	1.7	2.6	2.3			0.9	1.2	1.9	1.6		
Ave	2.5mg		4.0mg		12.5mg		0.9mg		1.7mg		4.4mg	
SD	.601		1.592		7.619		0.326		0.427		2.935	
P value			p=0.0020 vs CTL		p<0.0001 vs CTL p=0.0002 vs *FLExDUX4*				P<0.0001 vs CTL		P<0.0001 vs CTL P=0.0022 vs *FLExDUX4*	

S10 Fig. *DUX4-fl* expression leads to enlarged lymph nodes, suggesting a DUX4-mediated immune response. The subiliac and popliteal lymph nodes (2 of each from each mouse; NF = not found) were isolated from the indicated adult mice, cleared of attached tissues, and weighed. Both *FLExDUX4/+* and the *UBC-creERT2;FLExDUX4* mice, which expressed *DUX4-fl*, had significantly larger lymph nodes than control mice, which did not express *DUX4-fl*. In addition, the lymph nodes of *UBC-creERT2;FLExDUX4* mice were significantly larger than the those of *FLExDUX4/+* mice, correlating with the higher levels of functional DUX4-FL protein found in *UBC-creERT2;FLExDUX4* mice.
