# Supplementary material for: A cre-inducible DUX4 transgenic mouse model for investigating facioscapulohumeral muscular dystrophy
Source: PLoS One. 2018 Feb 7;13(2):e0192657. doi: 10.1371/journal.pone.0192657 (PMC5802938; doi:10.1371/journal.pone.0192657)
Supplement: S11 Fig — (PDF) [file pone.0192657.s013.pdf]

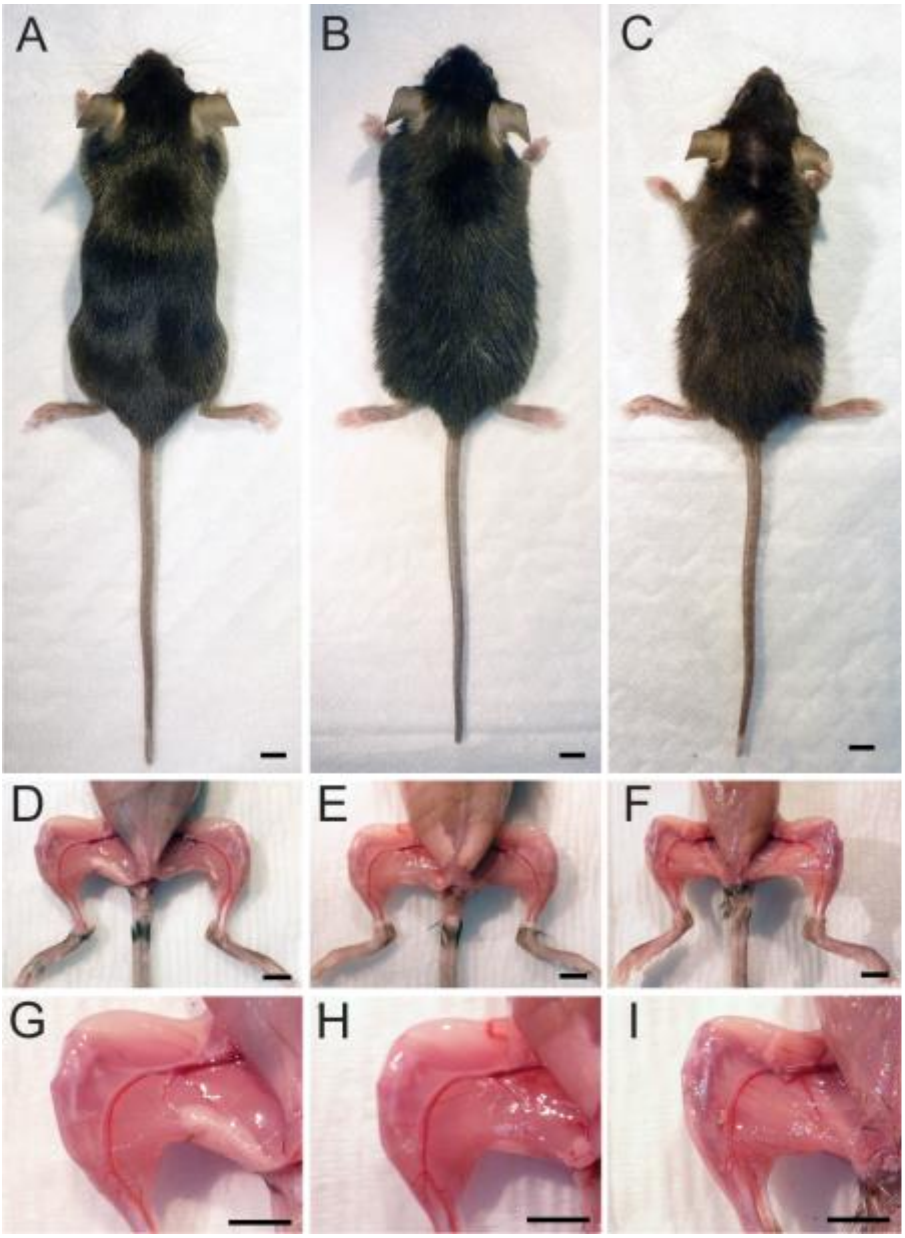

S11 Fig. TMX induction of DUX4-fl leads to physical decline and muscle loss in *ACTA1-MCM, FLExDUX4* mice. Mice (9 weeks old) were fed TMX-laced chow *ad libitum* for five consecutive days followed by 3 days of normal chow, and then sacrificed. TMX had no adverse effect on control *ACTA1-MCM* (A, D, G) or *FLExDUX4* (B, E, H) single transgenic mice. However, the *ACTA1-MCM;FLExDUX4* mice (C, F, I) drastically lost weight, muscle mass and mobility (S3 Movie). Scale bars = 5 mm.
