# Supplementary material for: A cre-inducible DUX4 transgenic mouse model for investigating facioscapulohumeral muscular dystrophy
Source: PLoS One. 2018 Feb 7;13(2):e0192657. doi: 10.1371/journal.pone.0192657 (PMC5802938; doi:10.1371/journal.pone.0192657)
Supplement: S12 Fig — (PDF) [file pone.0192657.s014.pdf]

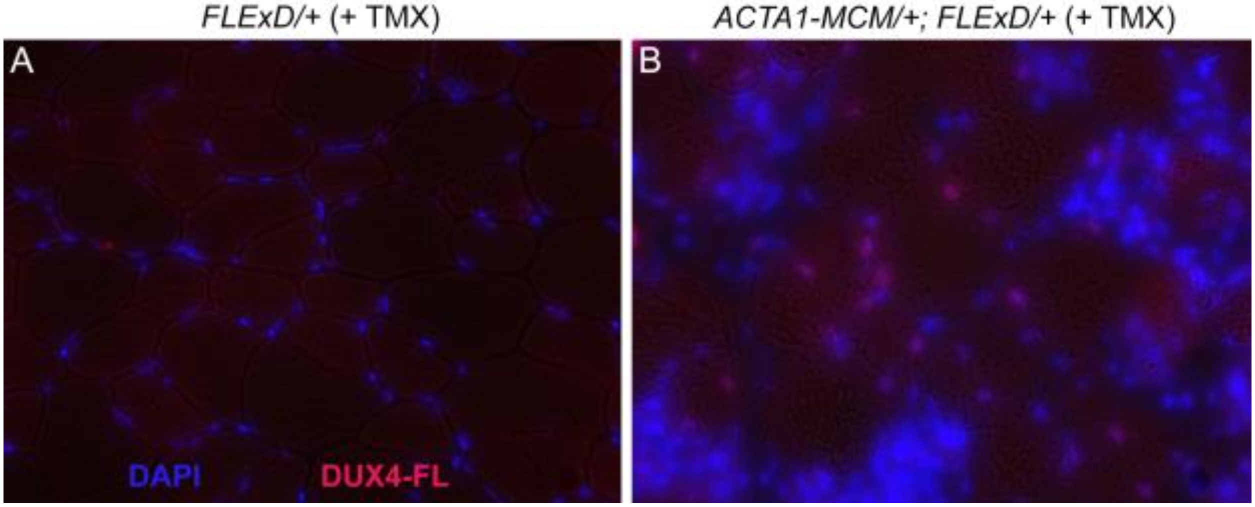

S12 Fig. TMX induces DUX4-FL protein and mononuclear cell infiltration in *ACTA1-MCM;FLExDUX4* double transgenic mice. A) *FLExDUX4*/+ and B) *ACTA1-MCM; FLExDUX4* were injected with TMX, and gastrocnemius muscles were analyzed 9 days post-injection by ICC for DUX4-FL protein expression (red) and counterstained with DAPI (blue) to visualize nuclei. Double transgenic mice showed a large increase in the number of myonuclei expressing DUX4-FL compared with the FLExDUX4/+ control; however, not all myonuclei were DUX4-FL positive. In addition, induced DUX4-FL expression correlated with a massive mononuclear cell infiltration.
