# Supplementary material for: A cre-inducible DUX4 transgenic mouse model for investigating facioscapulohumeral muscular dystrophy
Source: PLoS One. 2018 Feb 7;13(2):e0192657. doi: 10.1371/journal.pone.0192657 (PMC5802938; doi:10.1371/journal.pone.0192657)
Supplement: S13 Fig — (PDF) [file pone.0192657.s015.pdf]

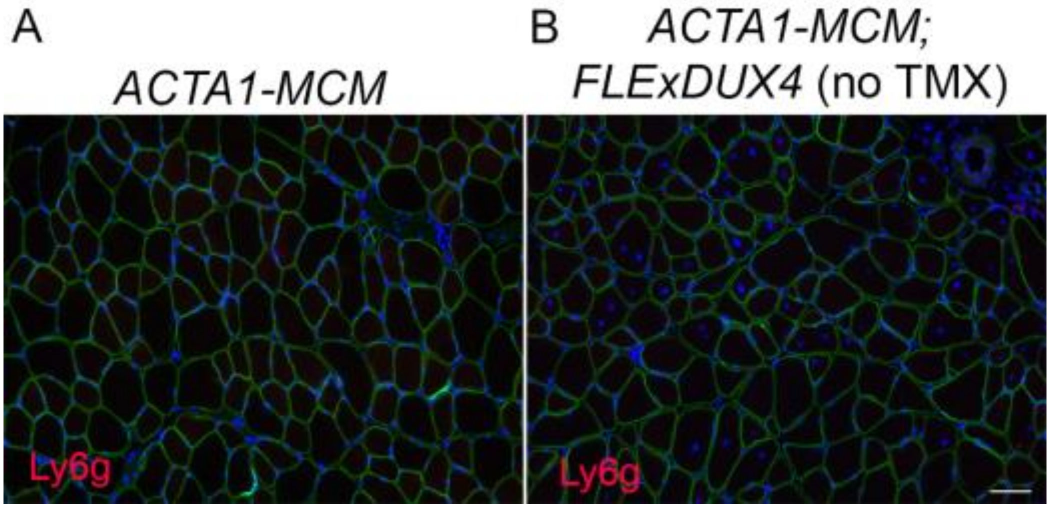

S13 Fig. Neutrophils do not accumulate in muscles of control mice. Control gastrocnemius muscle sections from A) *ACTA1-MCM* and B) *ACTA1-MCM;FLExDUX4* mice immunostained for Ly6g, a murine neutrophil marker. Supplemental data for Fig 14. Images are 20X, Bar = 25µm.
